# Supplementary figures and images for: Transferrin receptor regulates malignancies and the stemness of hepatocellular carcinoma-derived cancer stem-like cells by affecting iron accumulation
Source: PLoS One. 2020 Dec 22;15(12):e0243812. doi: 10.1371/journal.pone.0243812 (PMC7755206; doi:10.1371/journal.pone.0243812)

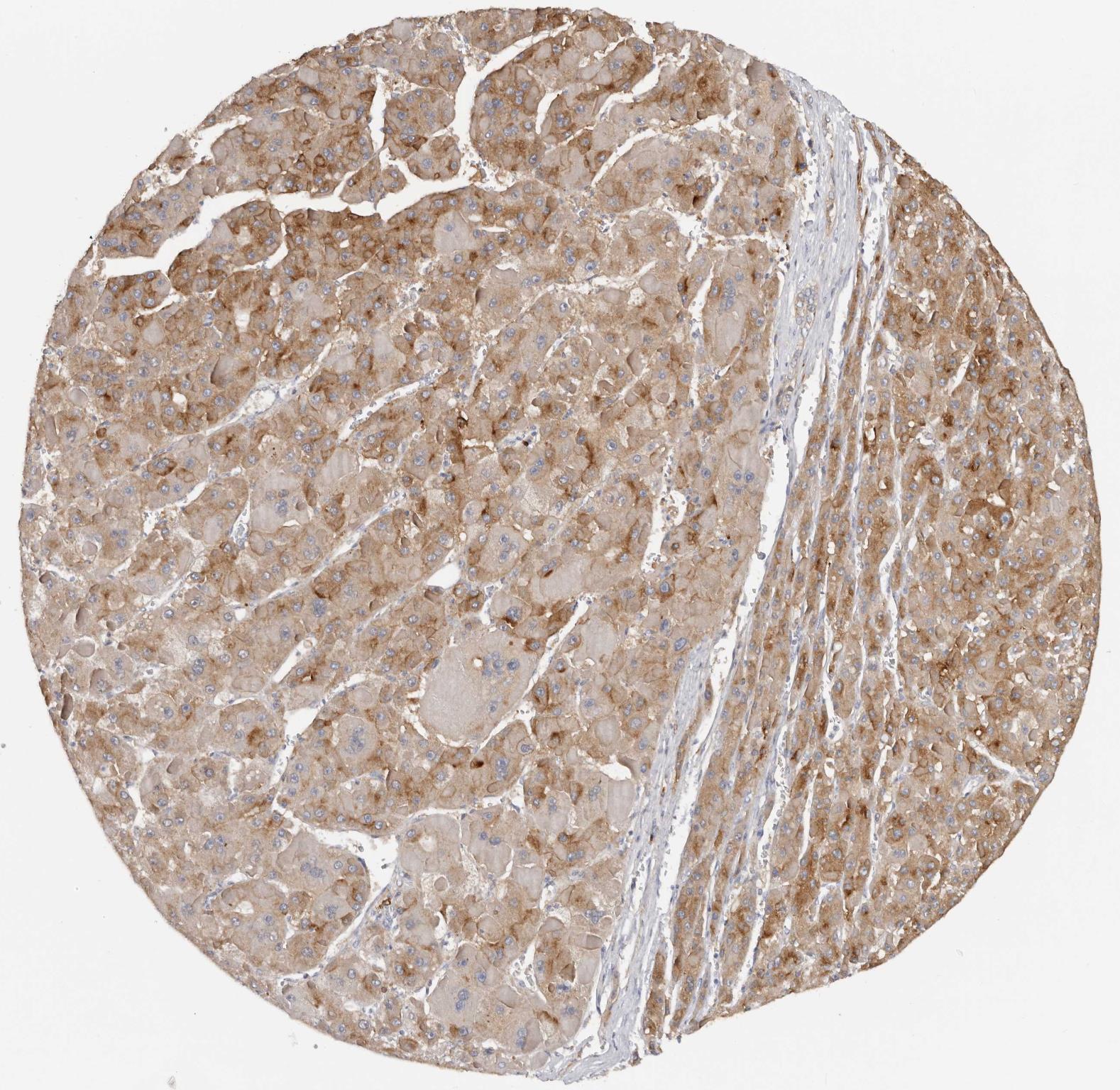

Supplement: S1 File — (ZIP) [file pone.0243812.s001.zip › supporting information/figure 1d. LHIC.jpg]

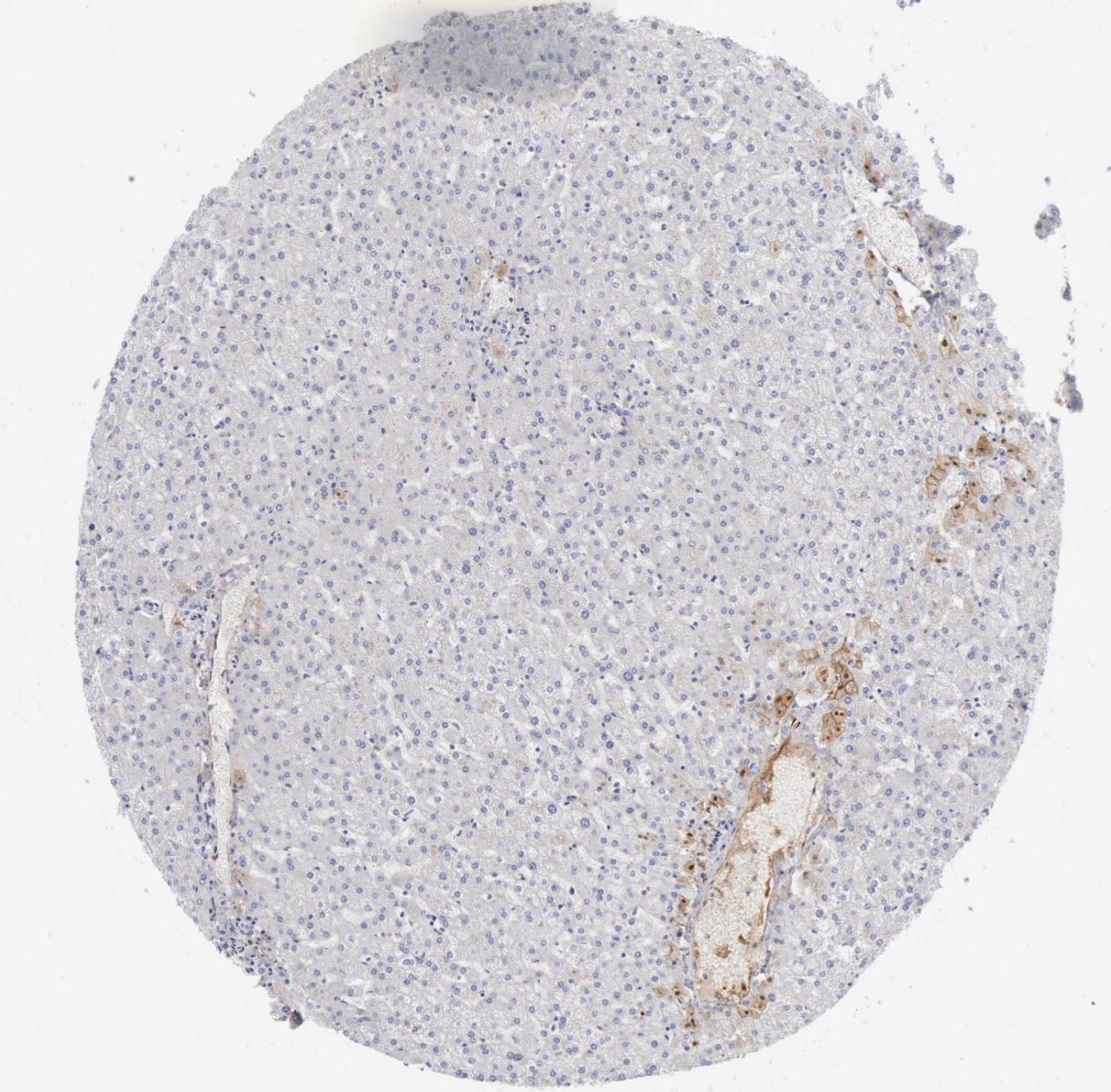

Supplement: S1 File — (ZIP) [file pone.0243812.s001.zip › supporting information/figure 1d. LIVER.jpg]

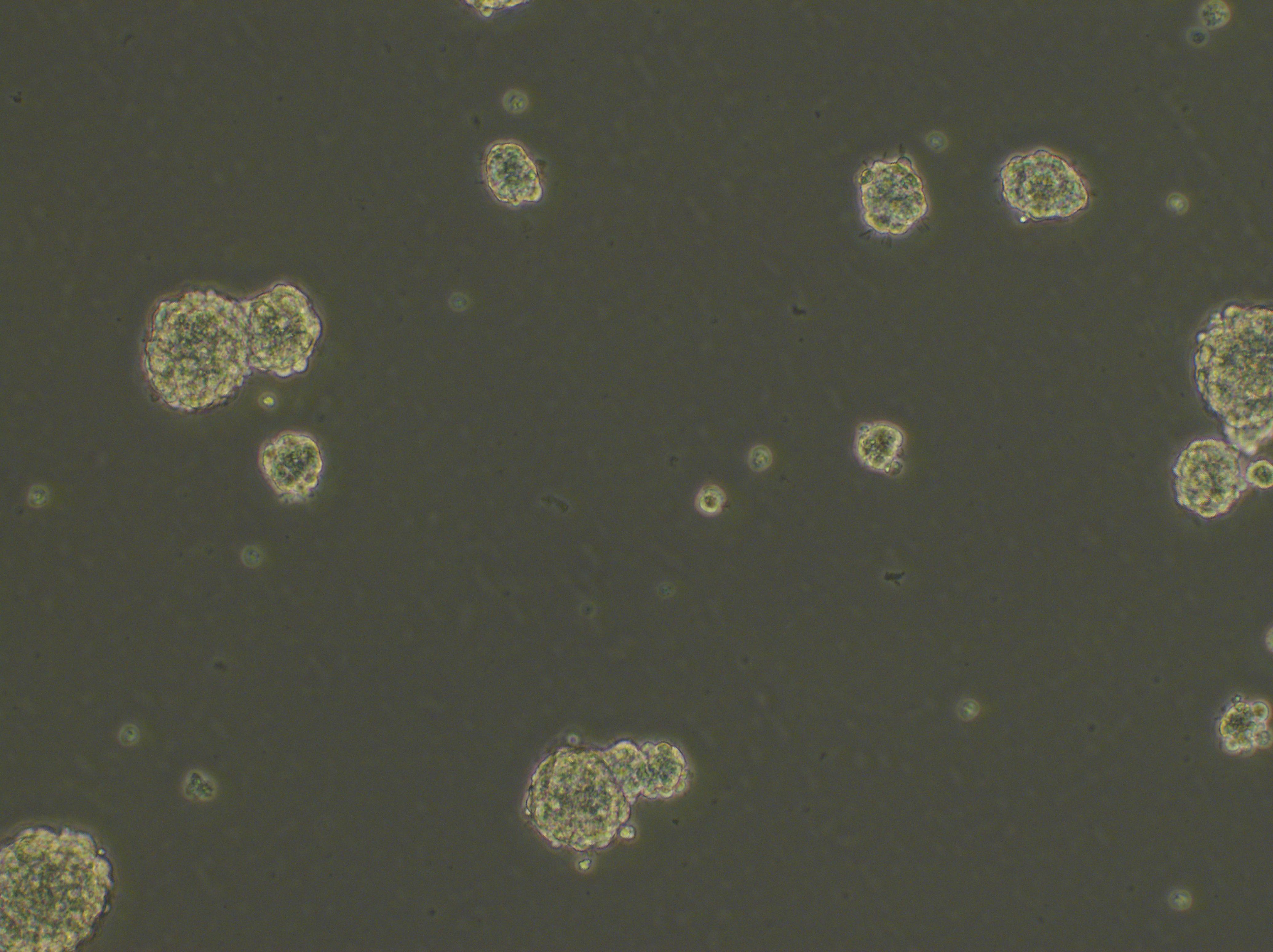

Supplement: S1 File — (ZIP) [file pone.0243812.s001.zip › supporting information/figure 2a-1.jpg]

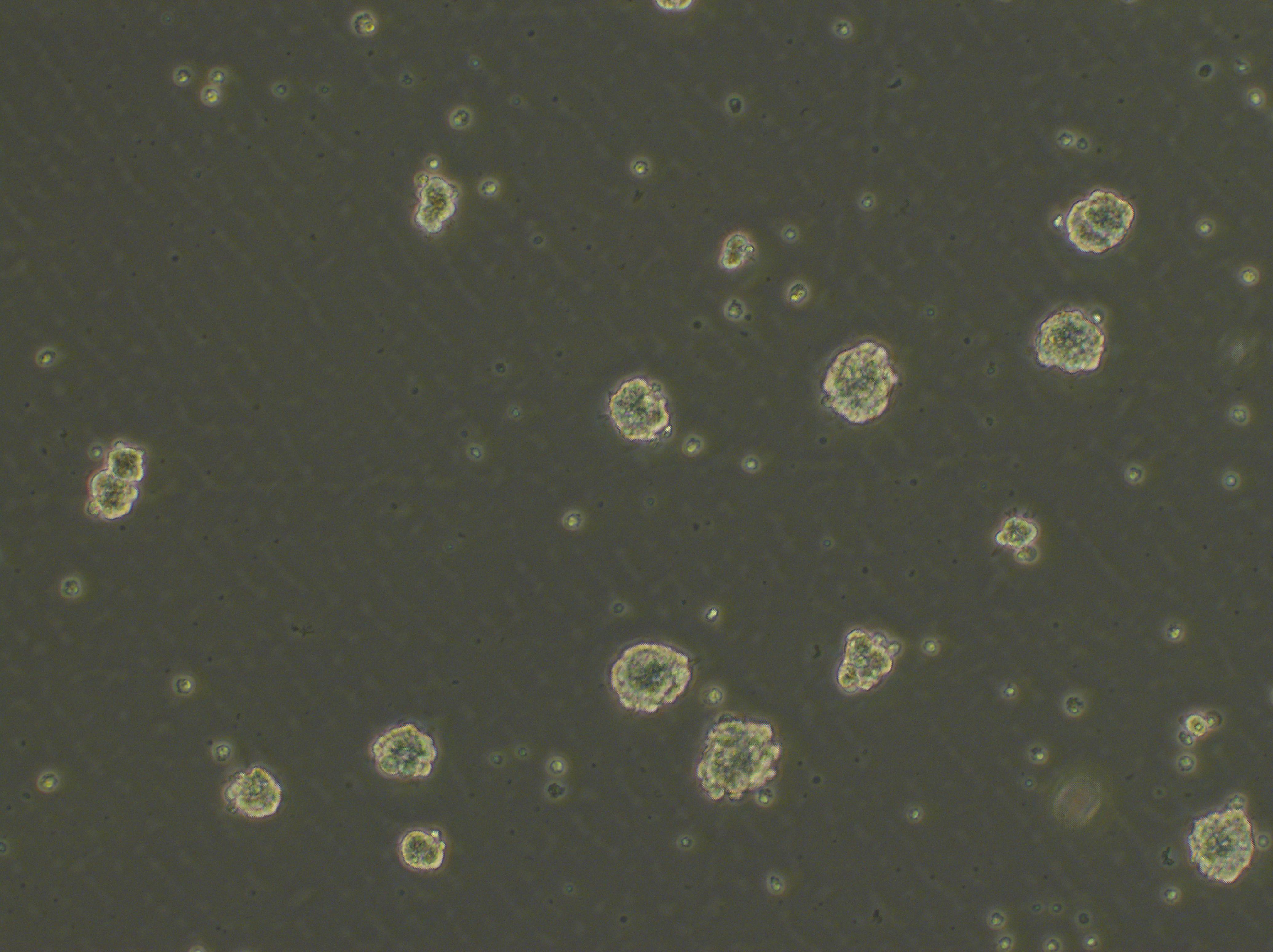

Supplement: S1 File — (ZIP) [file pone.0243812.s001.zip › supporting information/figure 2a-2.jpg]

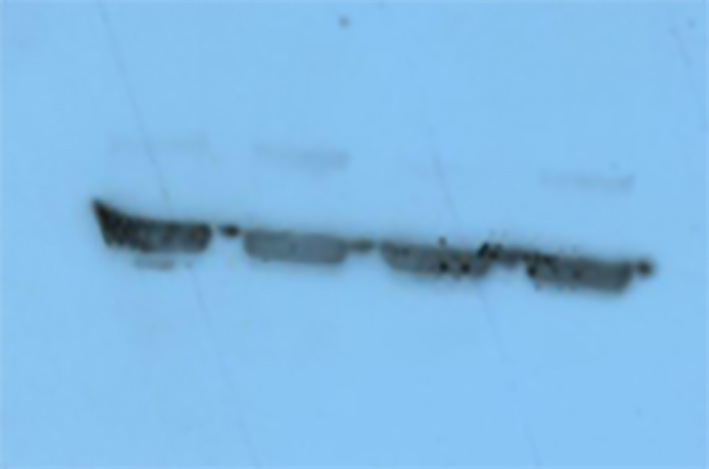

Supplement: S1 File — (ZIP) [file pone.0243812.s001.zip › supporting information/figure 2b actin.jpg]

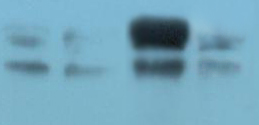

Supplement: S1 File — (ZIP) [file pone.0243812.s001.zip › supporting information/figure 2b.jpg]

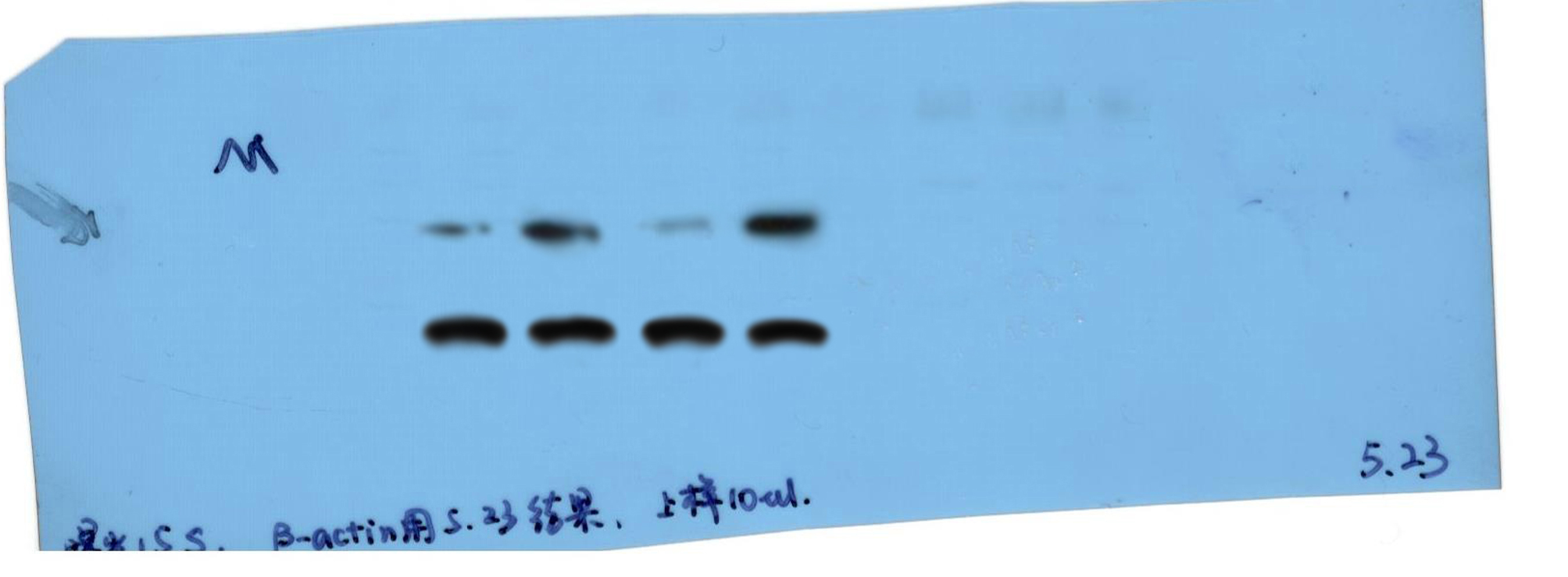

Supplement: S1 File — (ZIP) [file pone.0243812.s001.zip › supporting information/figure 2c, original blots.jpg]

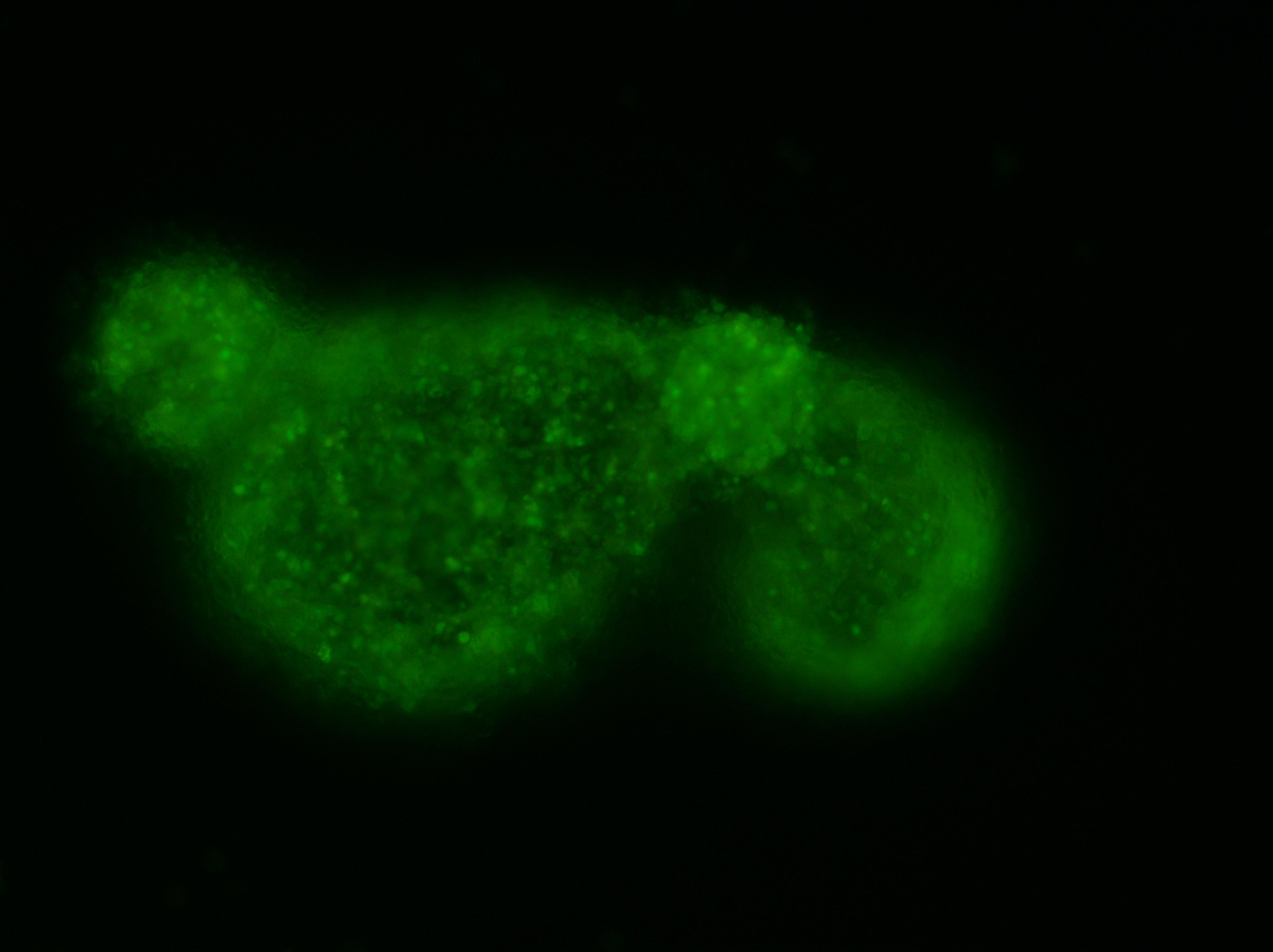

Supplement: S1 File — (ZIP) [file pone.0243812.s001.zip › supporting information/figure 3a Huh-7/1.jpg]

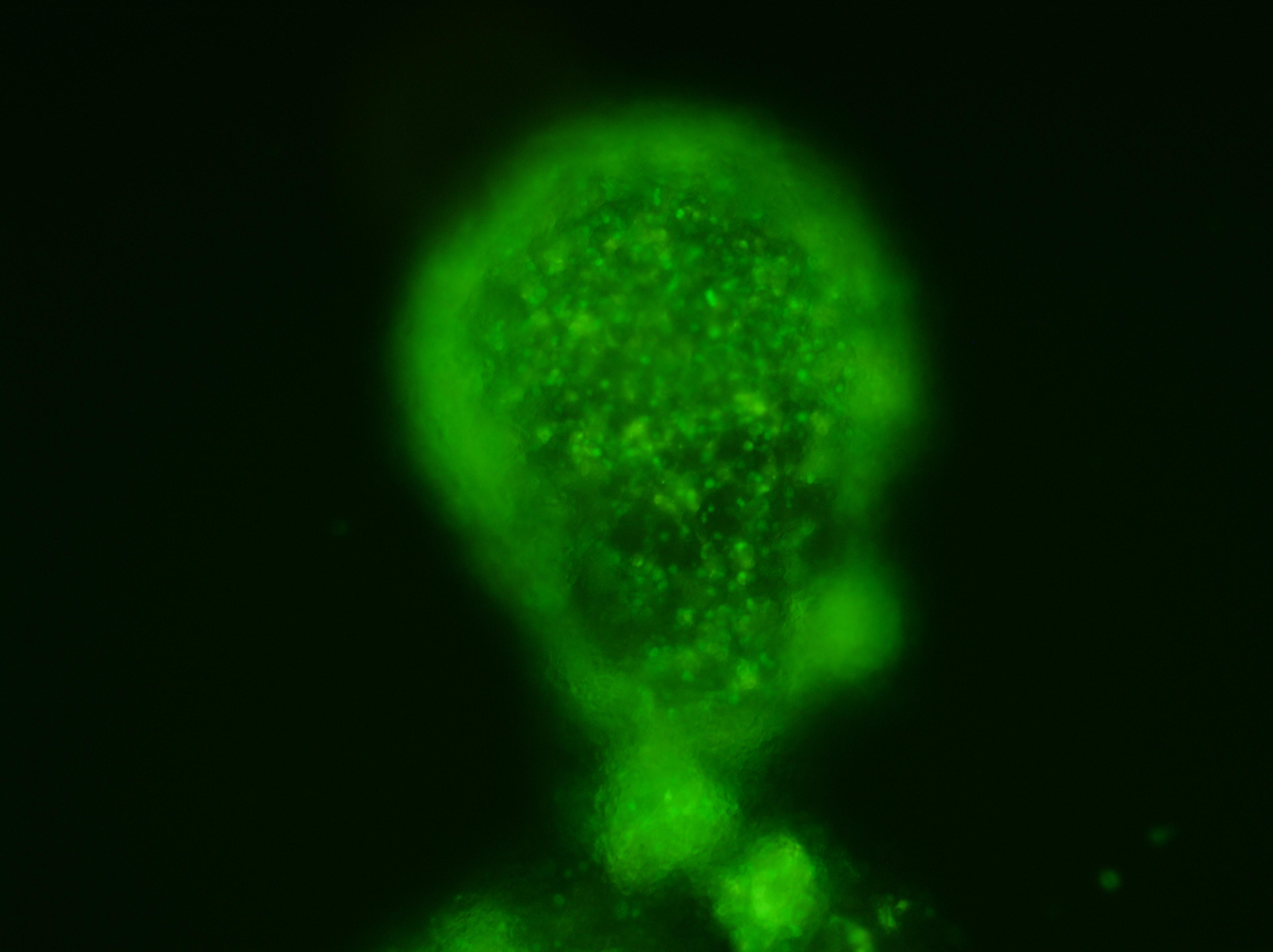

Supplement: S1 File — (ZIP) [file pone.0243812.s001.zip › supporting information/figure 3a Huh-7/2.jpg]

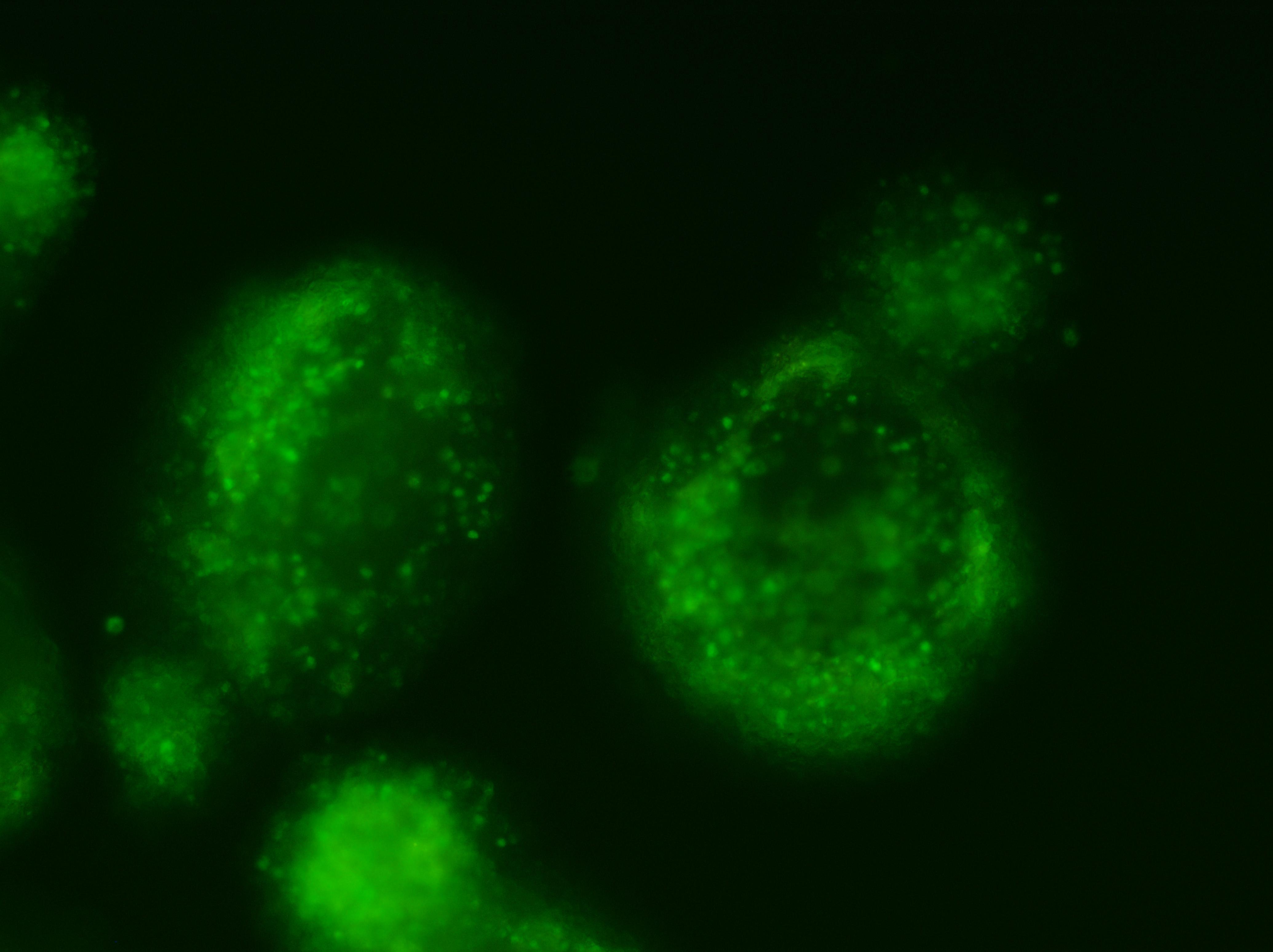

Supplement: S1 File — (ZIP) [file pone.0243812.s001.zip › supporting information/figure 3a Huh-7/3.jpg]

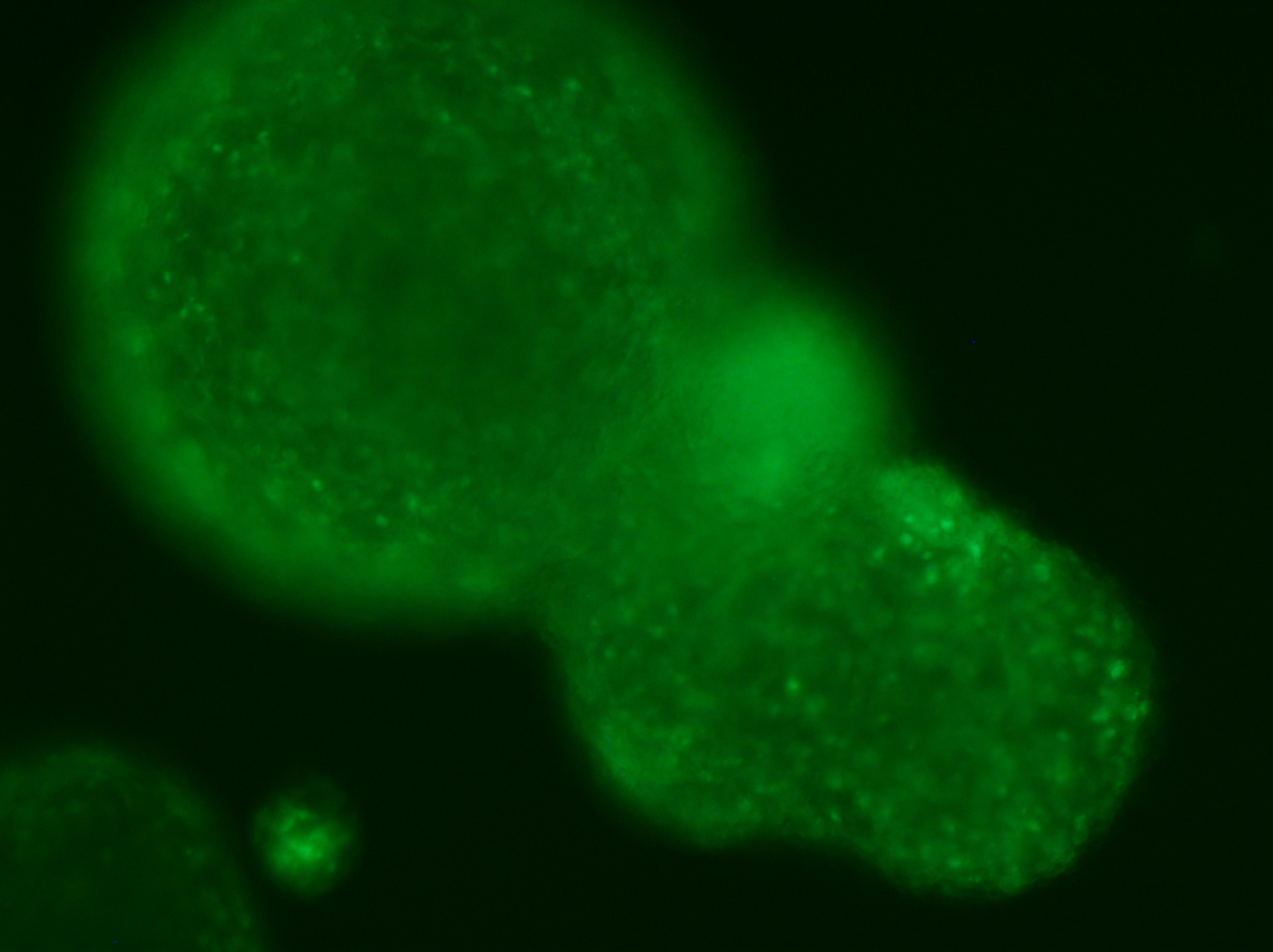

Supplement: S1 File — (ZIP) [file pone.0243812.s001.zip › supporting information/figure 3a SK-HEP-1/1.jpg]

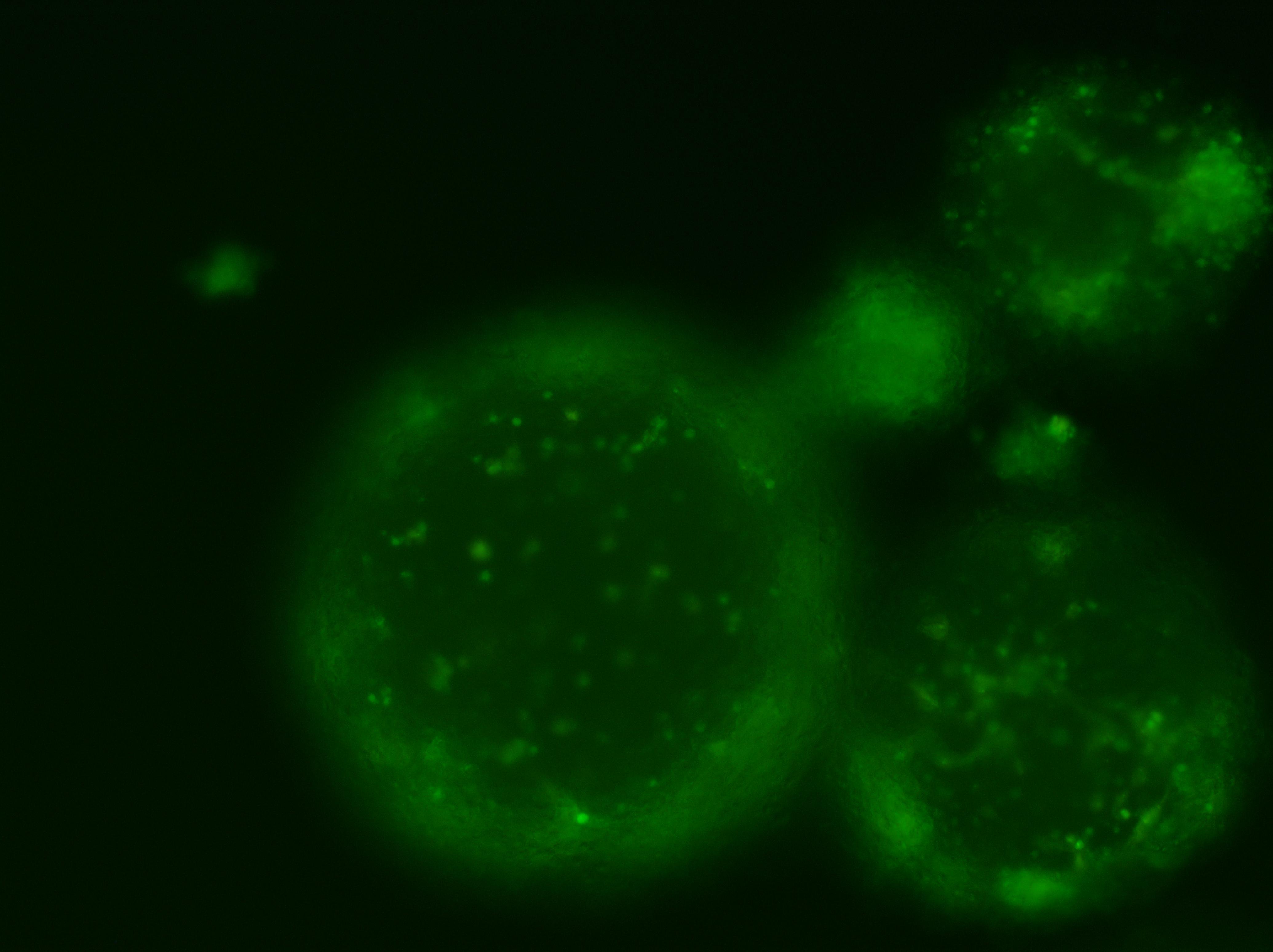

Supplement: S1 File — (ZIP) [file pone.0243812.s001.zip › supporting information/figure 3a SK-HEP-1/2.jpg]

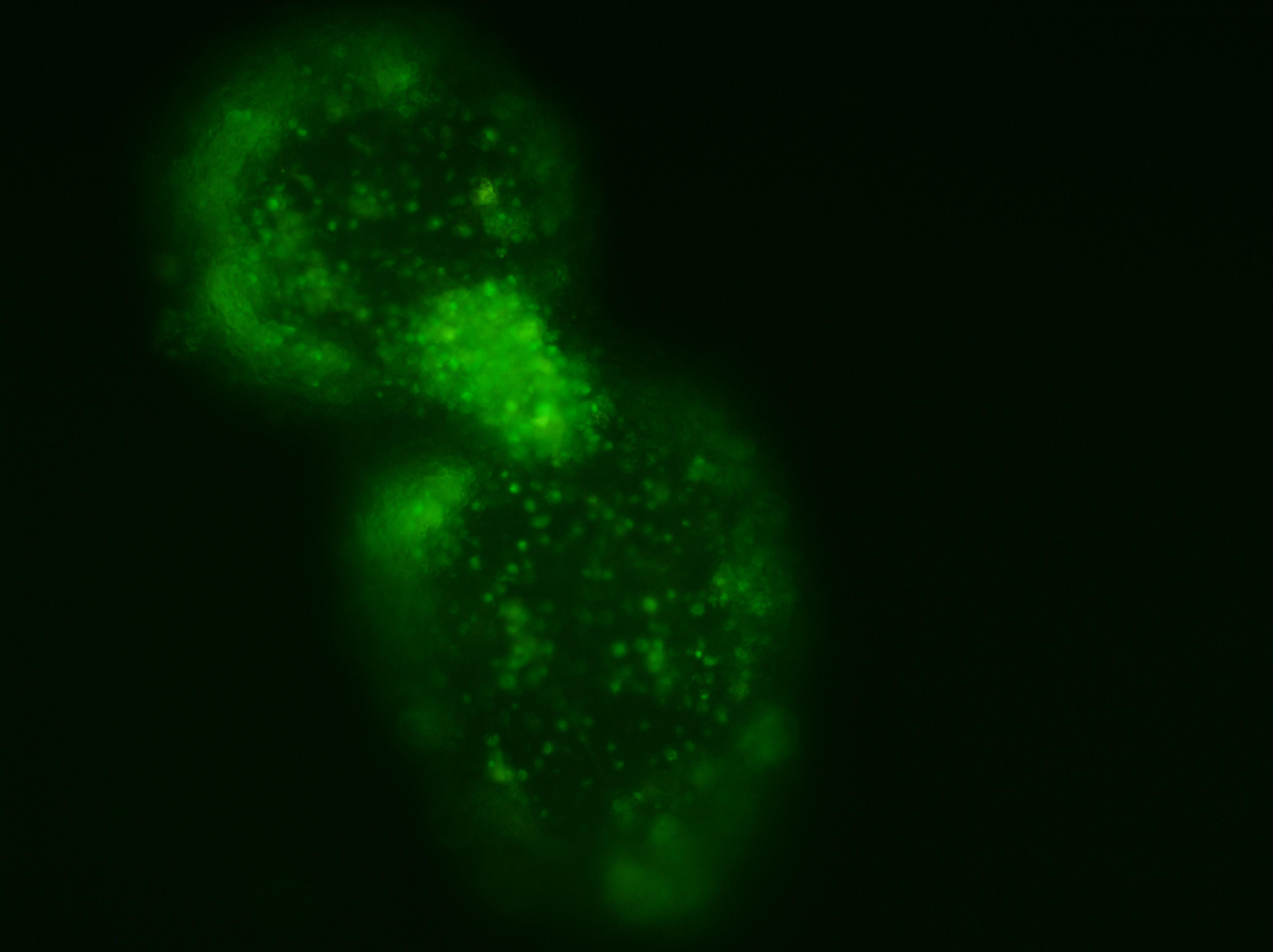

Supplement: S1 File — (ZIP) [file pone.0243812.s001.zip › supporting information/figure 3a SK-HEP-1/3.jpg]

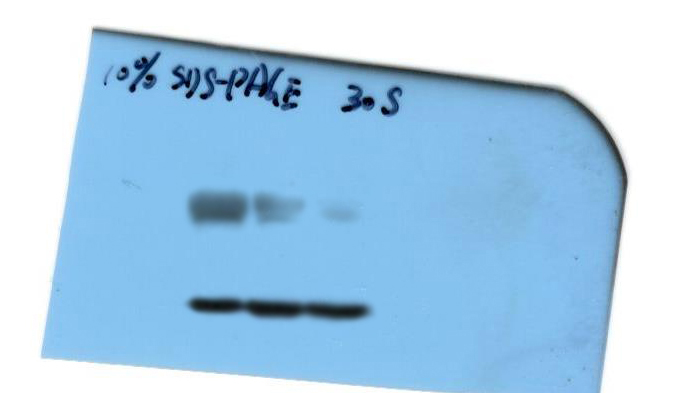

Supplement: S1 File — (ZIP) [file pone.0243812.s001.zip › supporting information/figure 3b-1.jpg]

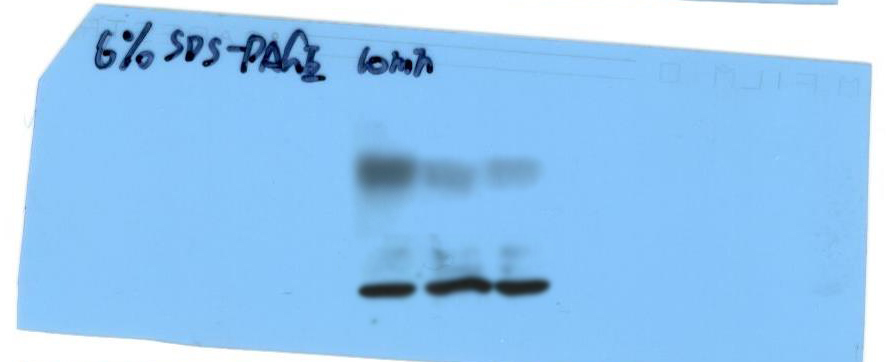

Supplement: S1 File — (ZIP) [file pone.0243812.s001.zip › supporting information/figure 3b-2.jpg]

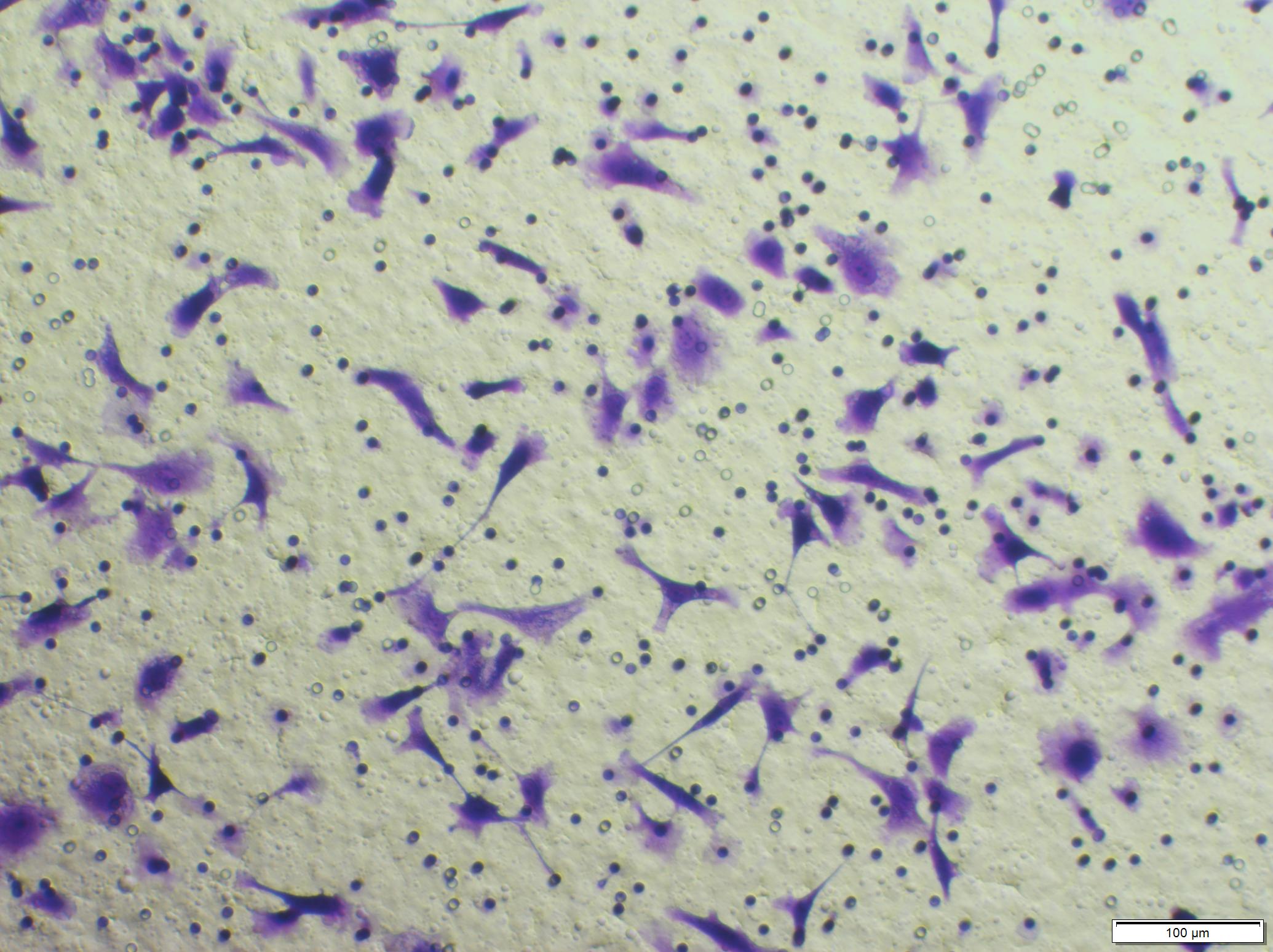

Supplement: S1 File — (ZIP) [file pone.0243812.s001.zip › supporting information/figure 3d-1.jpg]

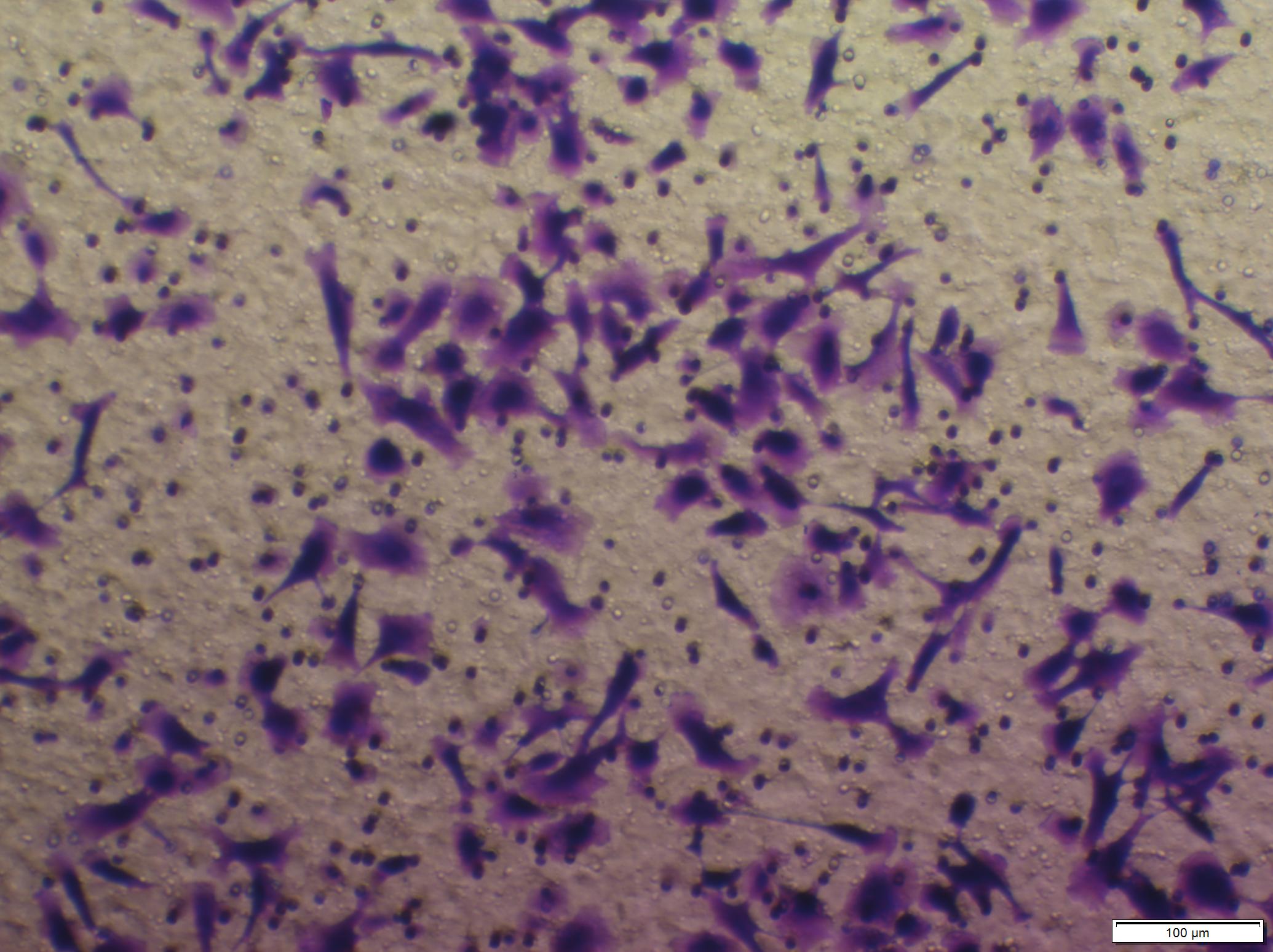

Supplement: S1 File — (ZIP) [file pone.0243812.s001.zip › supporting information/figure 3d-2.jpg]

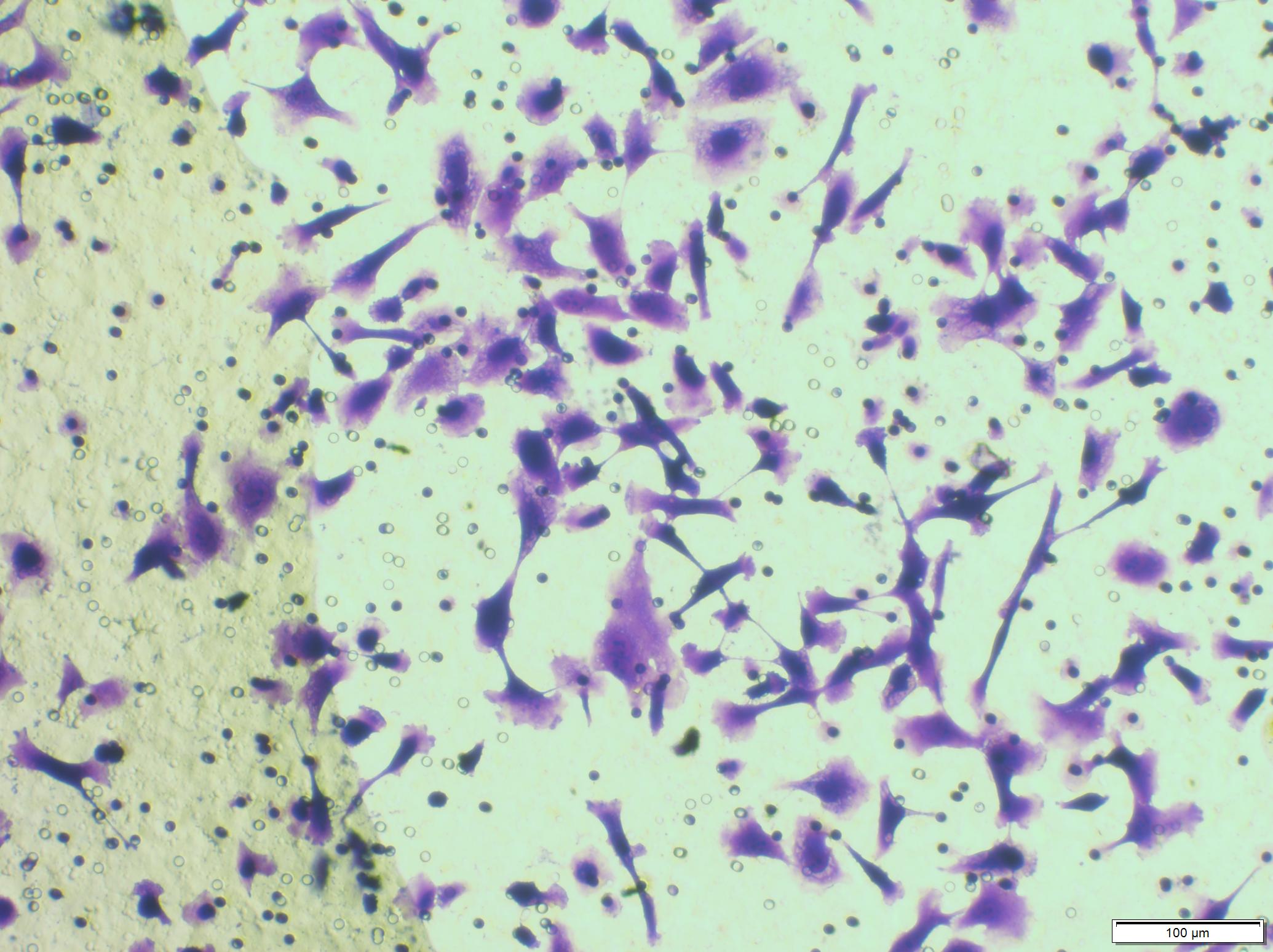

Supplement: S1 File — (ZIP) [file pone.0243812.s001.zip › supporting information/figure 3d-3.jpg]

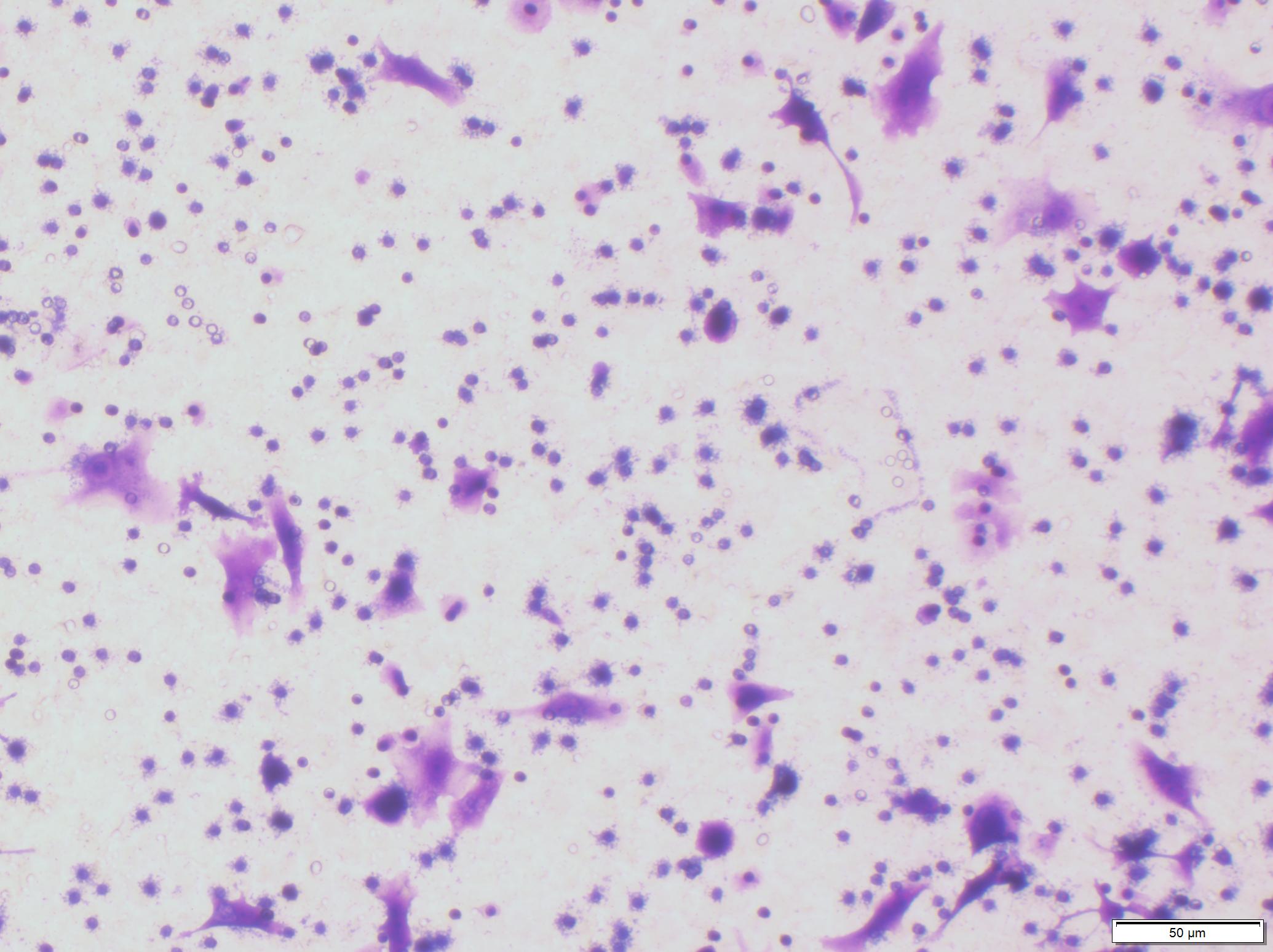

Supplement: S1 File — (ZIP) [file pone.0243812.s001.zip › supporting information/figure 3d-4.jpg]

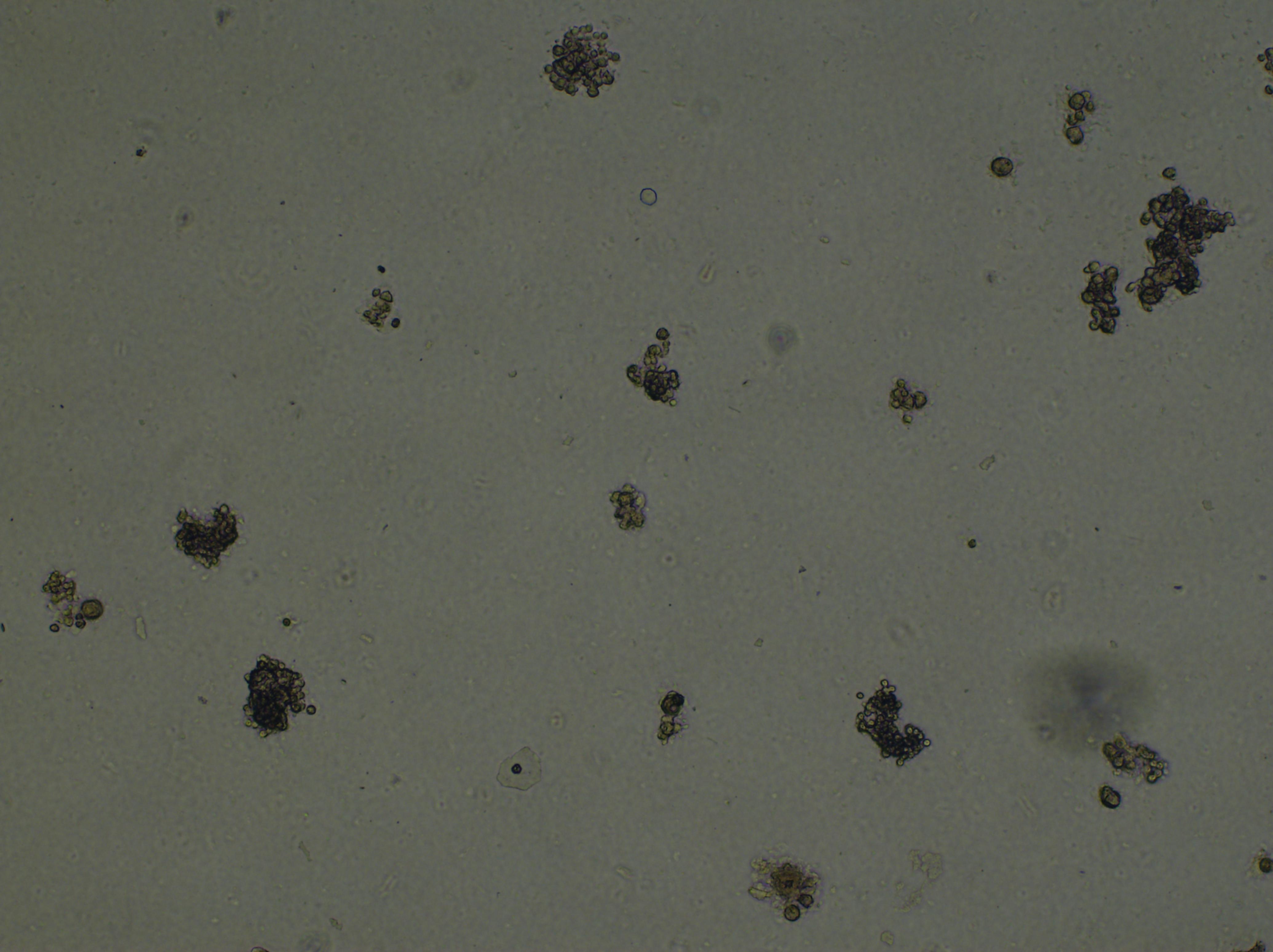

Supplement: S1 File — (ZIP) [file pone.0243812.s001.zip › supporting information/figure 3e-1.jpg]

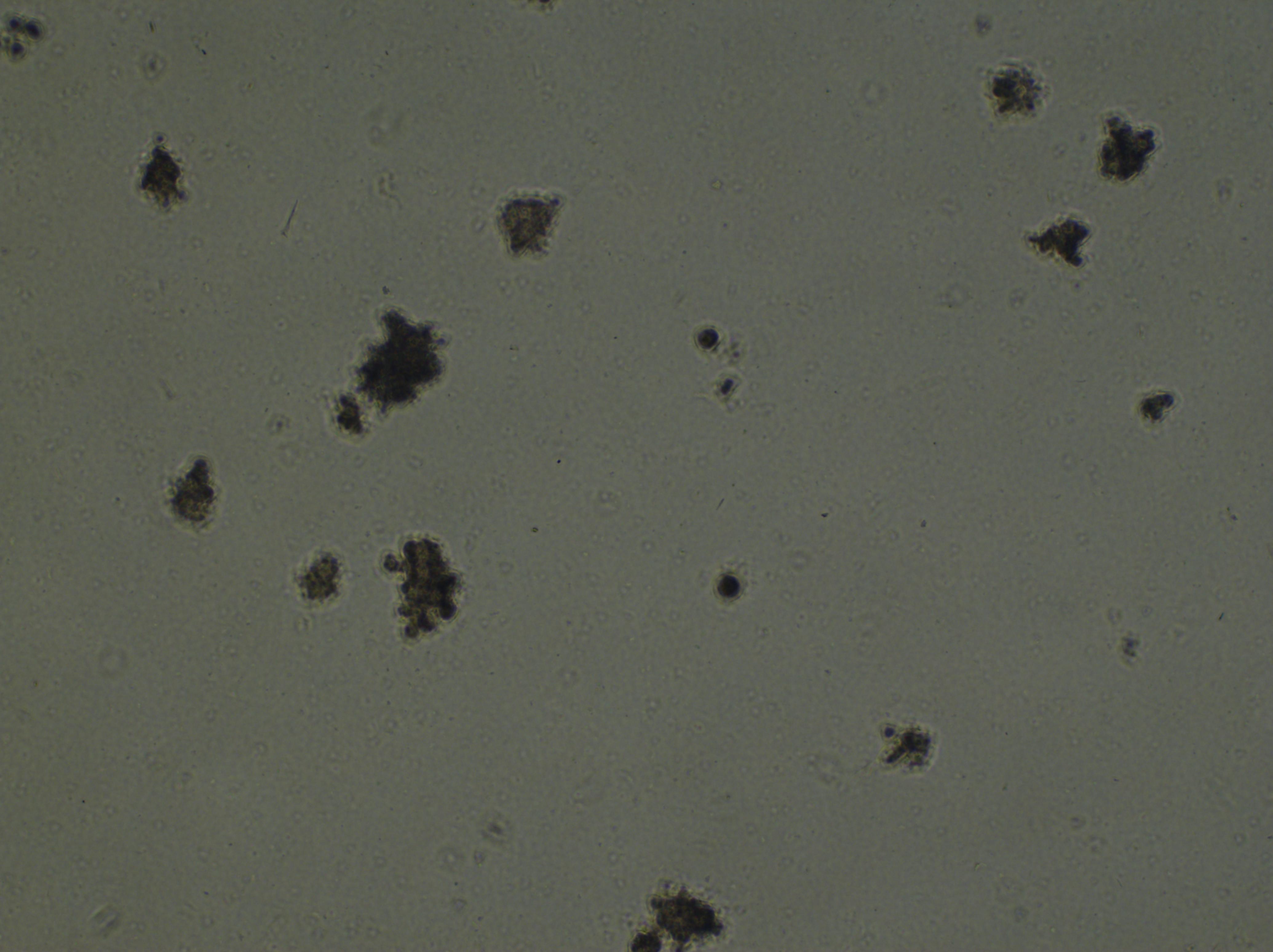

Supplement: S1 File — (ZIP) [file pone.0243812.s001.zip › supporting information/figure 3e-2.jpg]

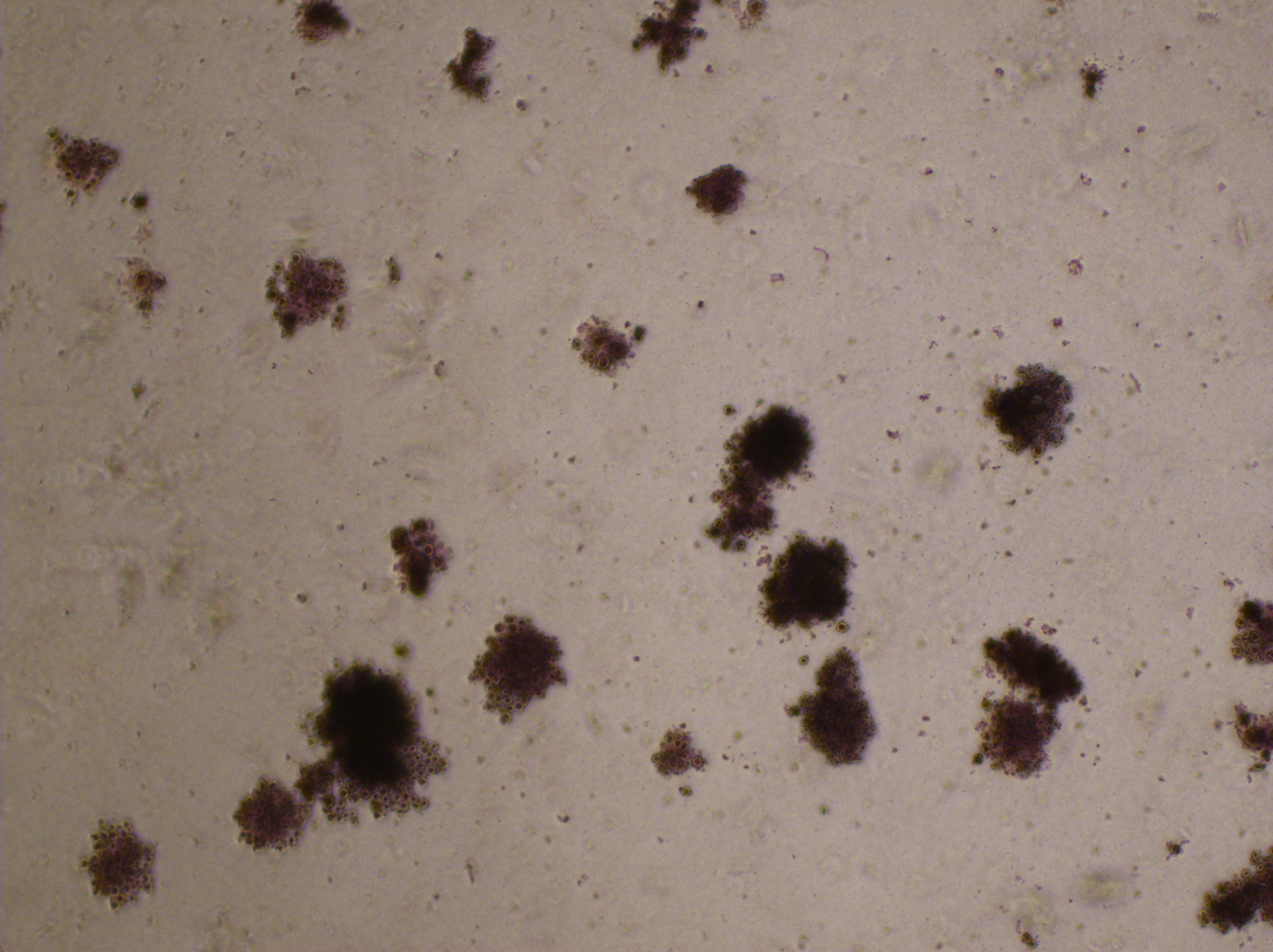

Supplement: S1 File — (ZIP) [file pone.0243812.s001.zip › supporting information/figure 3e-3.jpg]

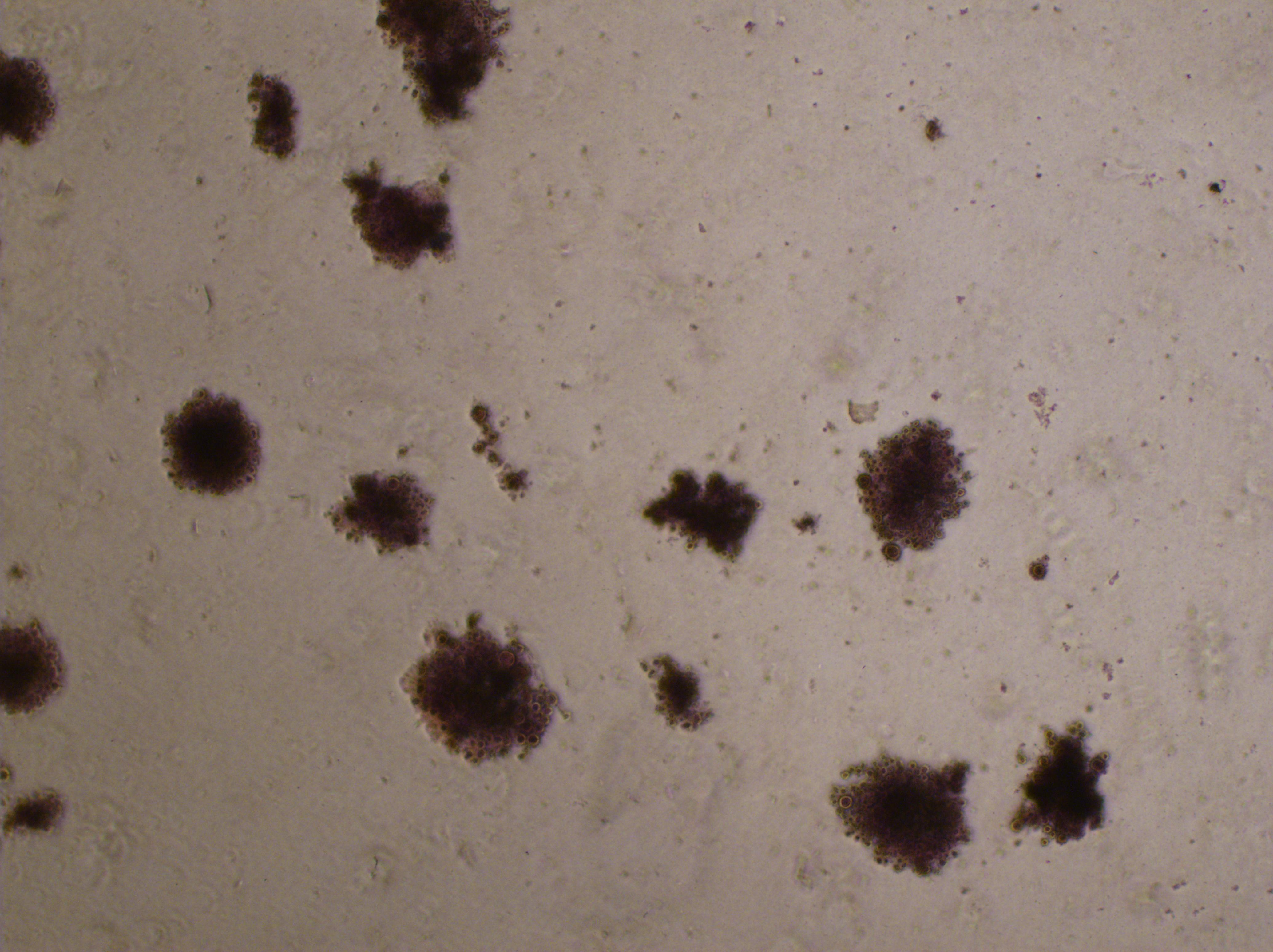

Supplement: S1 File — (ZIP) [file pone.0243812.s001.zip › supporting information/figure 3e-4.jpg]

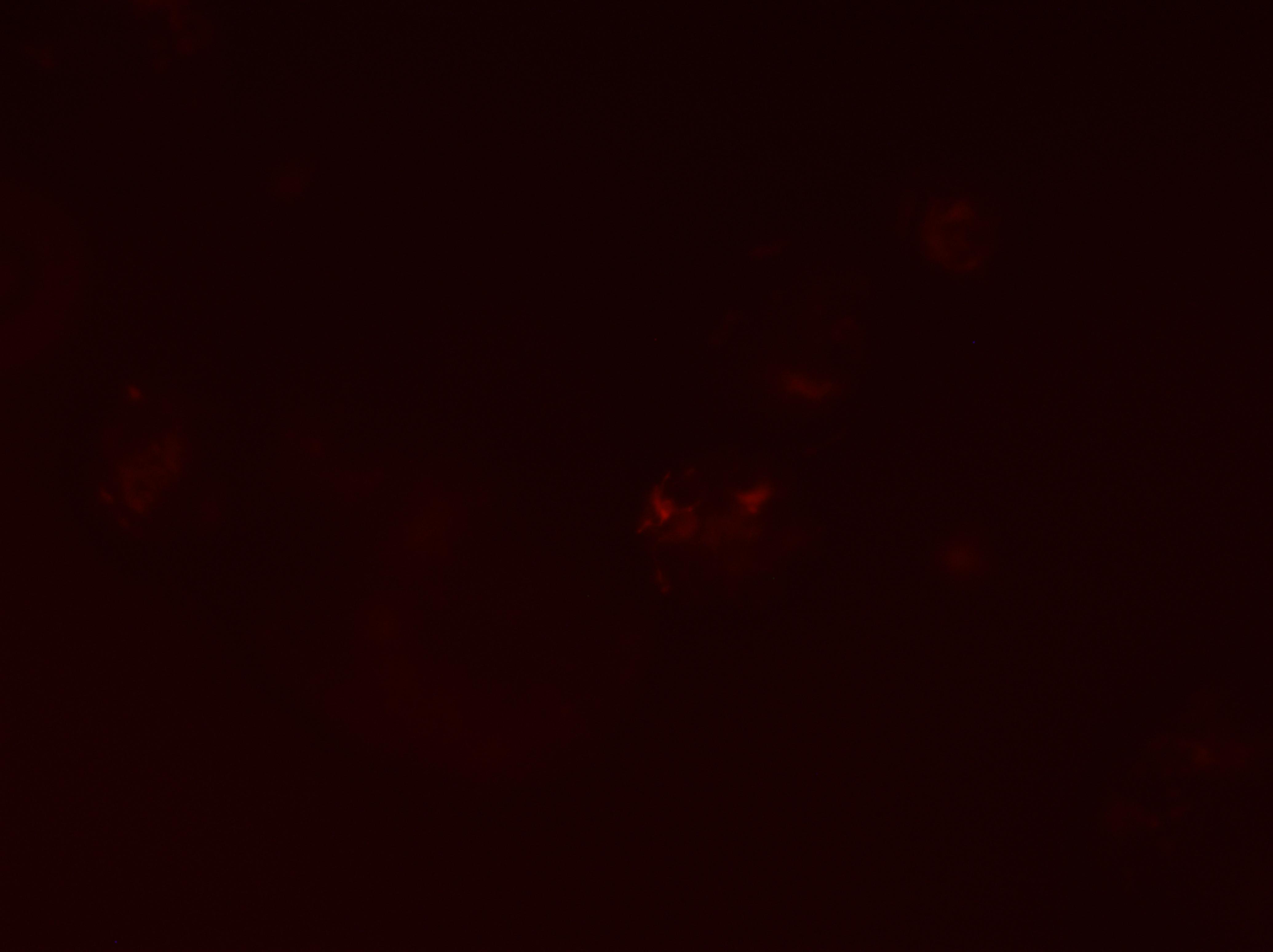

Supplement: S1 File — (ZIP) [file pone.0243812.s001.zip › supporting information/figure 4c-1.jpg]

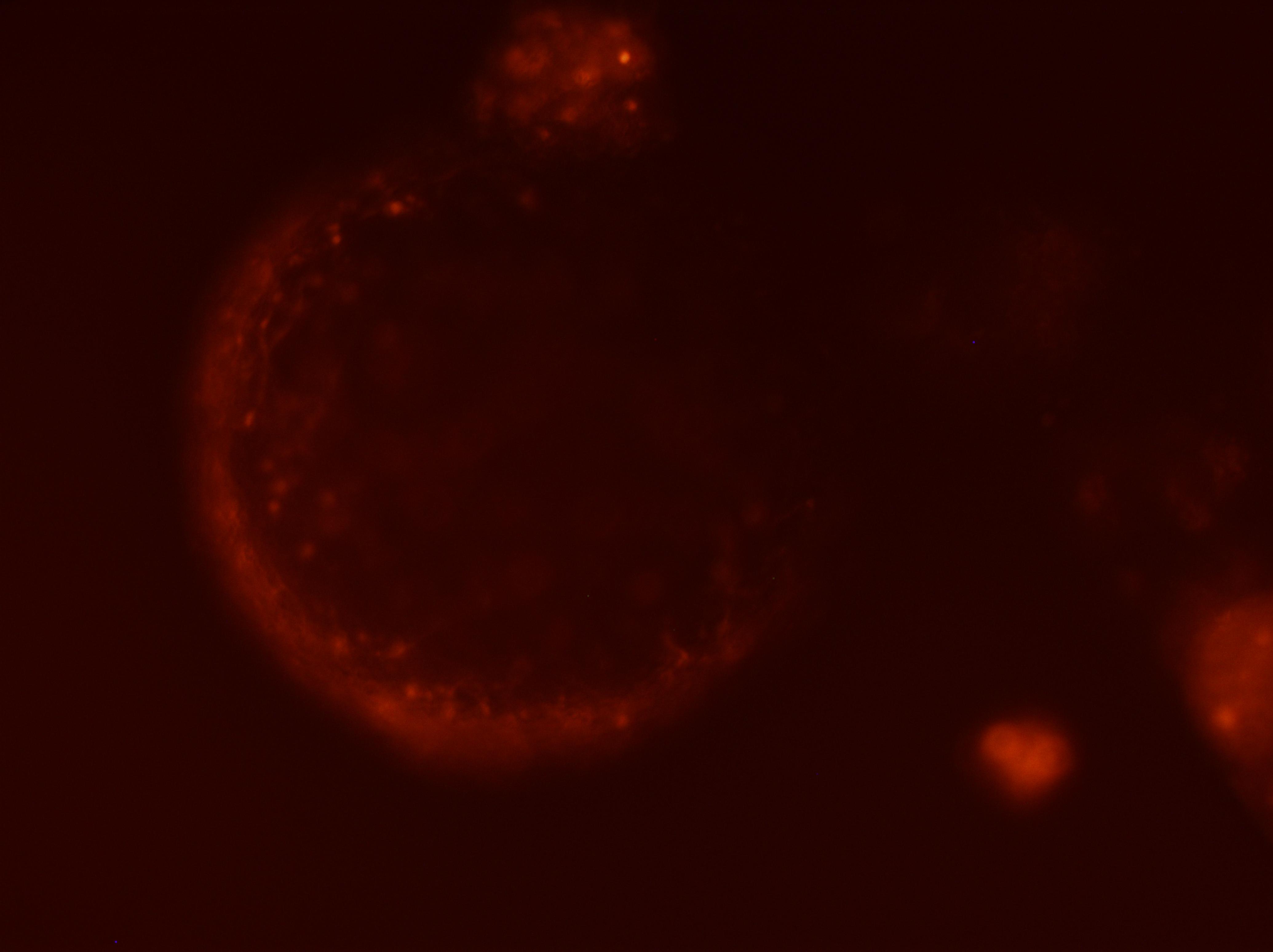

Supplement: S1 File — (ZIP) [file pone.0243812.s001.zip › supporting information/figure 4c-2.jpg]

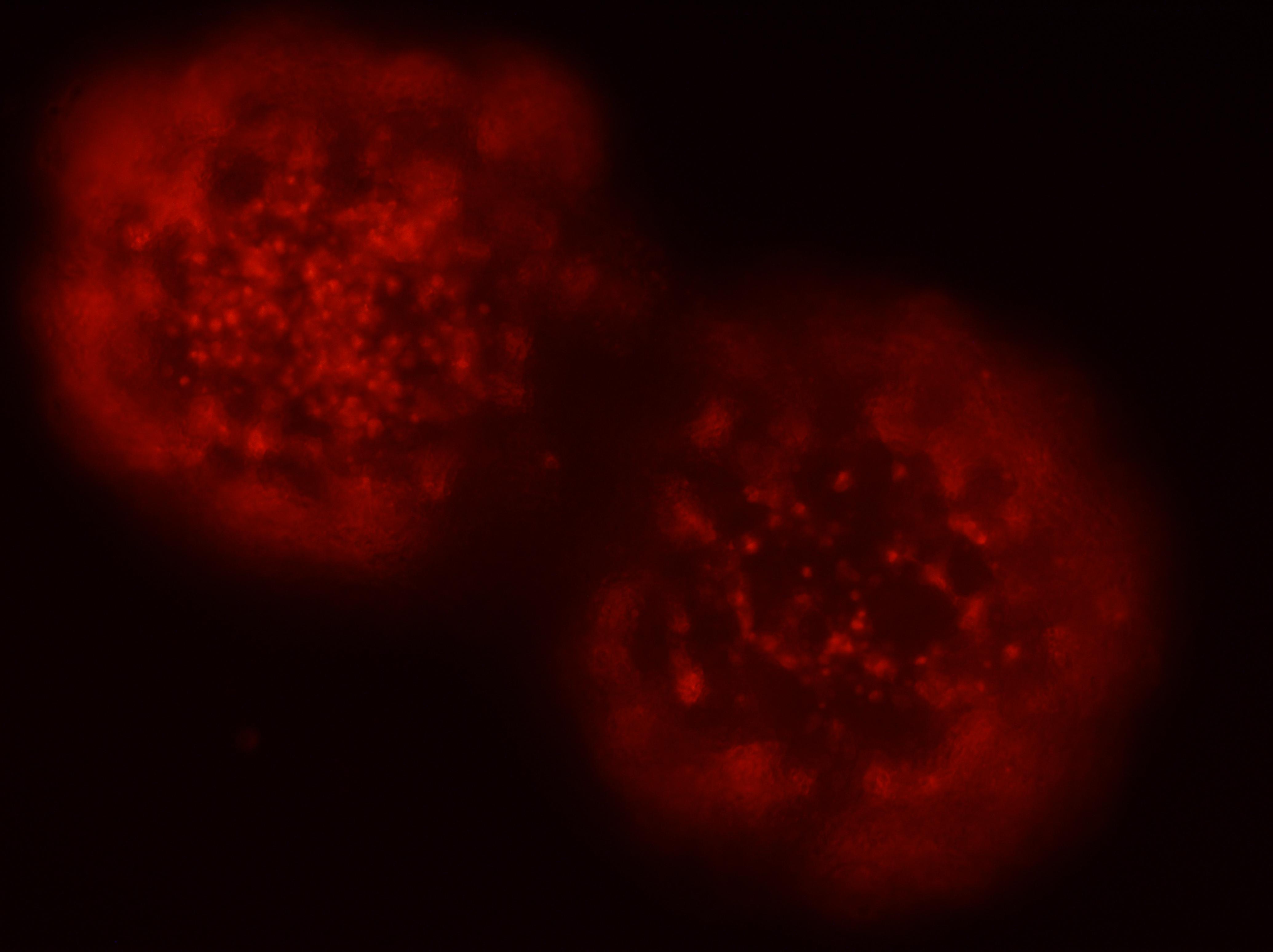

Supplement: S1 File — (ZIP) [file pone.0243812.s001.zip › supporting information/figure 4c-3.jpg]

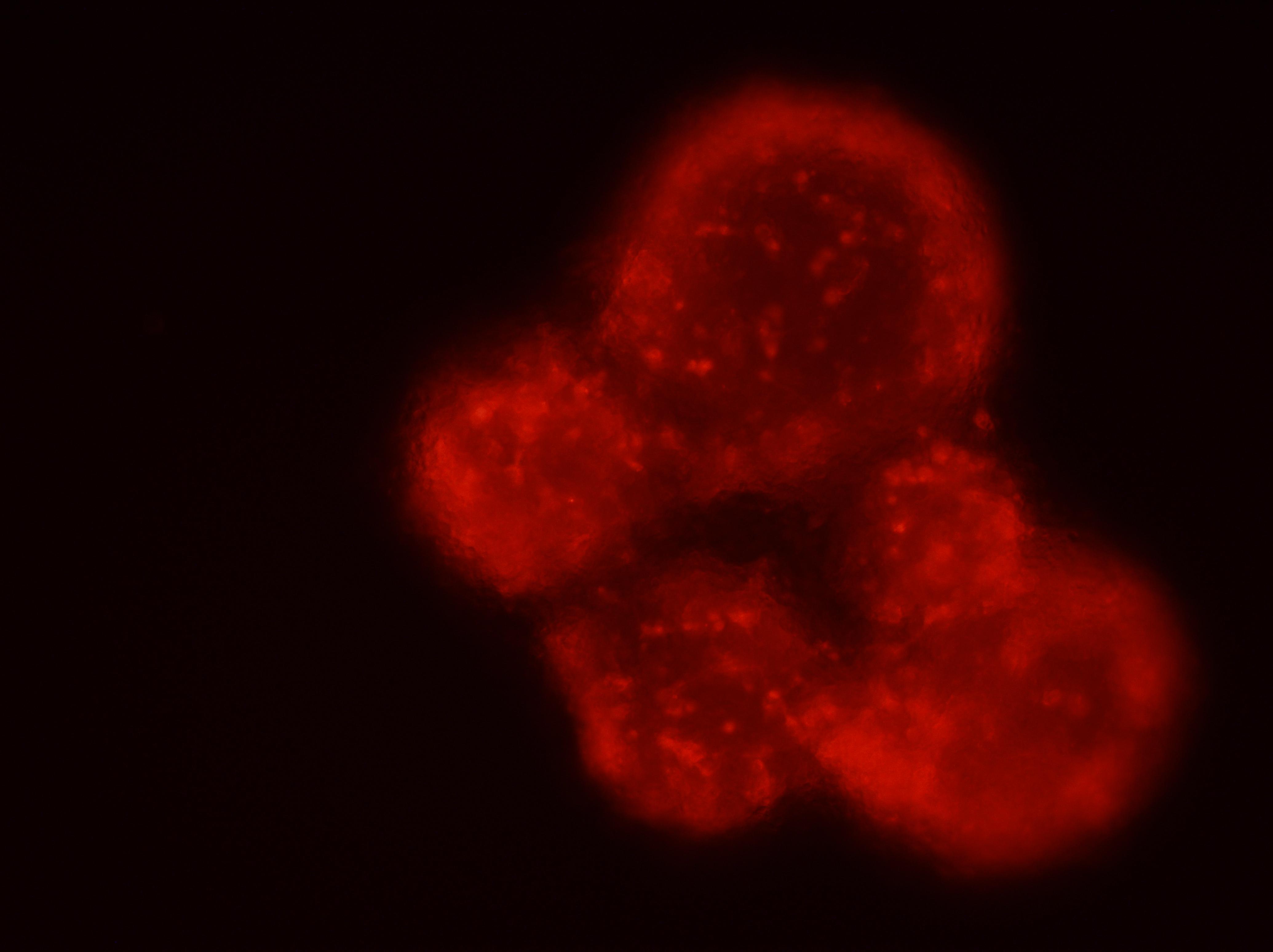

Supplement: S1 File — (ZIP) [file pone.0243812.s001.zip › supporting information/figure 4c-4.jpg]

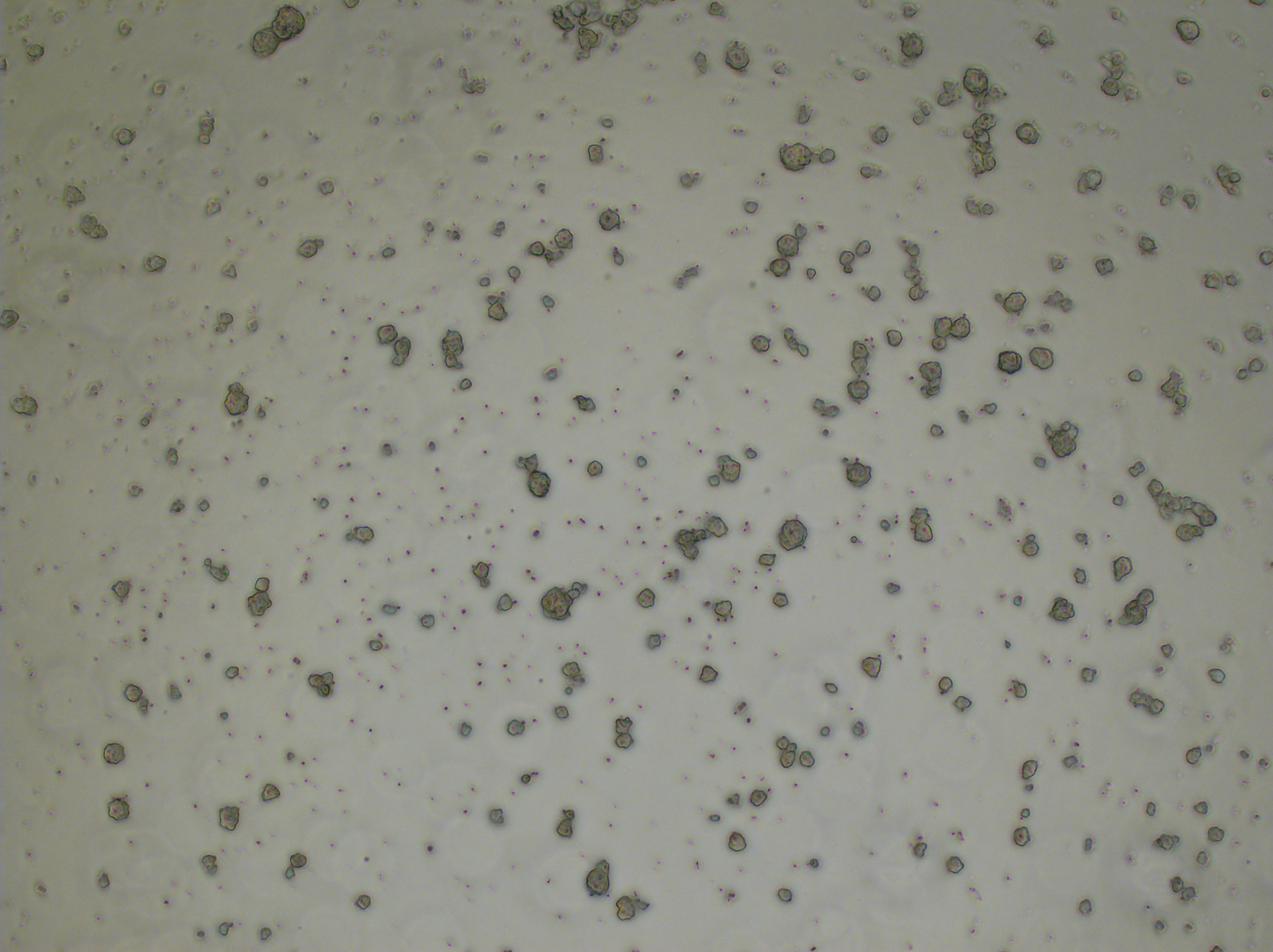

Supplement: S1 File — (ZIP) [file pone.0243812.s001.zip › supporting information/figure 5a/LEFT PANEL/40▒╢-1.jpg]

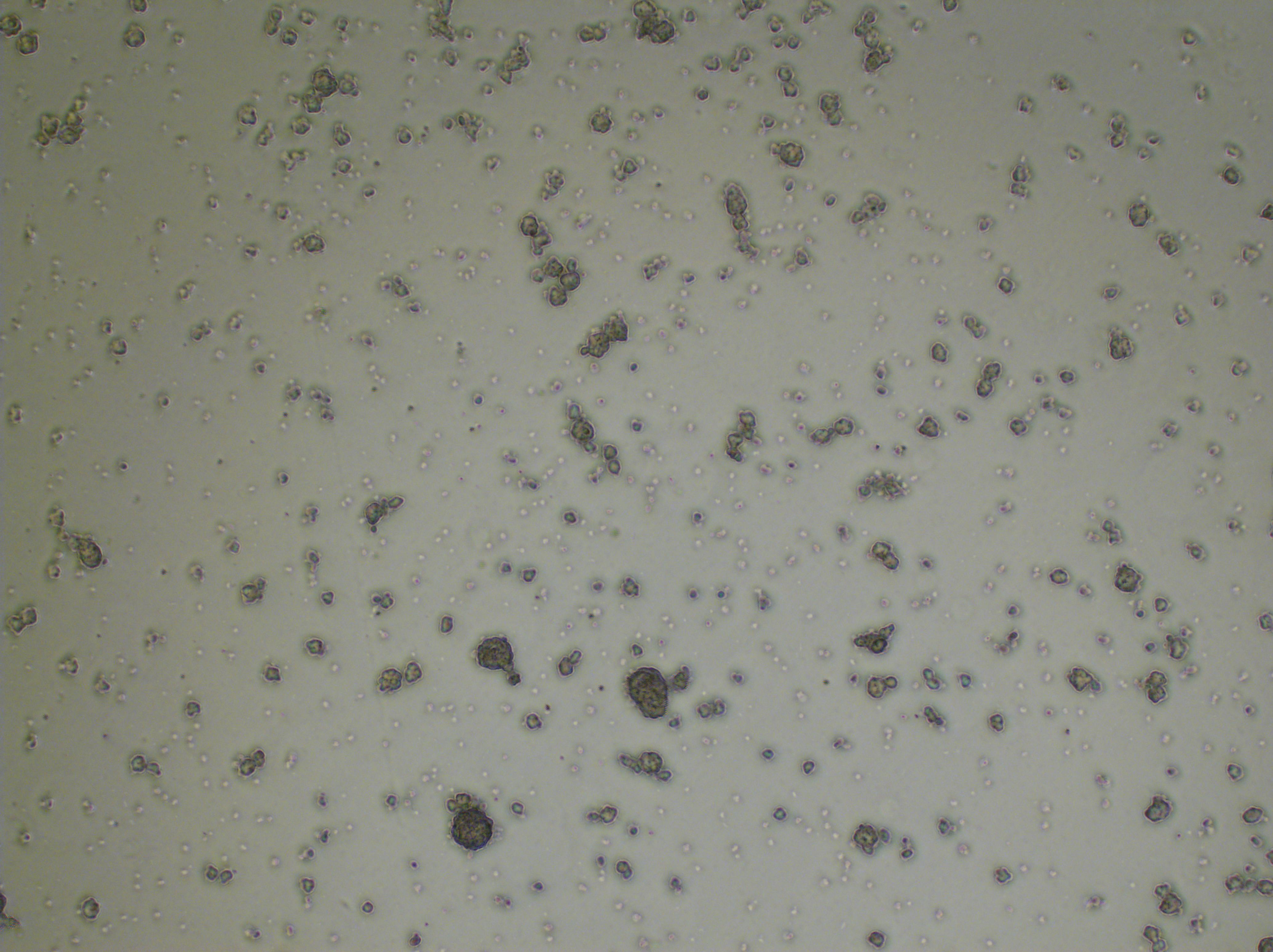

Supplement: S1 File — (ZIP) [file pone.0243812.s001.zip › supporting information/figure 5a/LEFT PANEL/40▒╢-2.jpg]

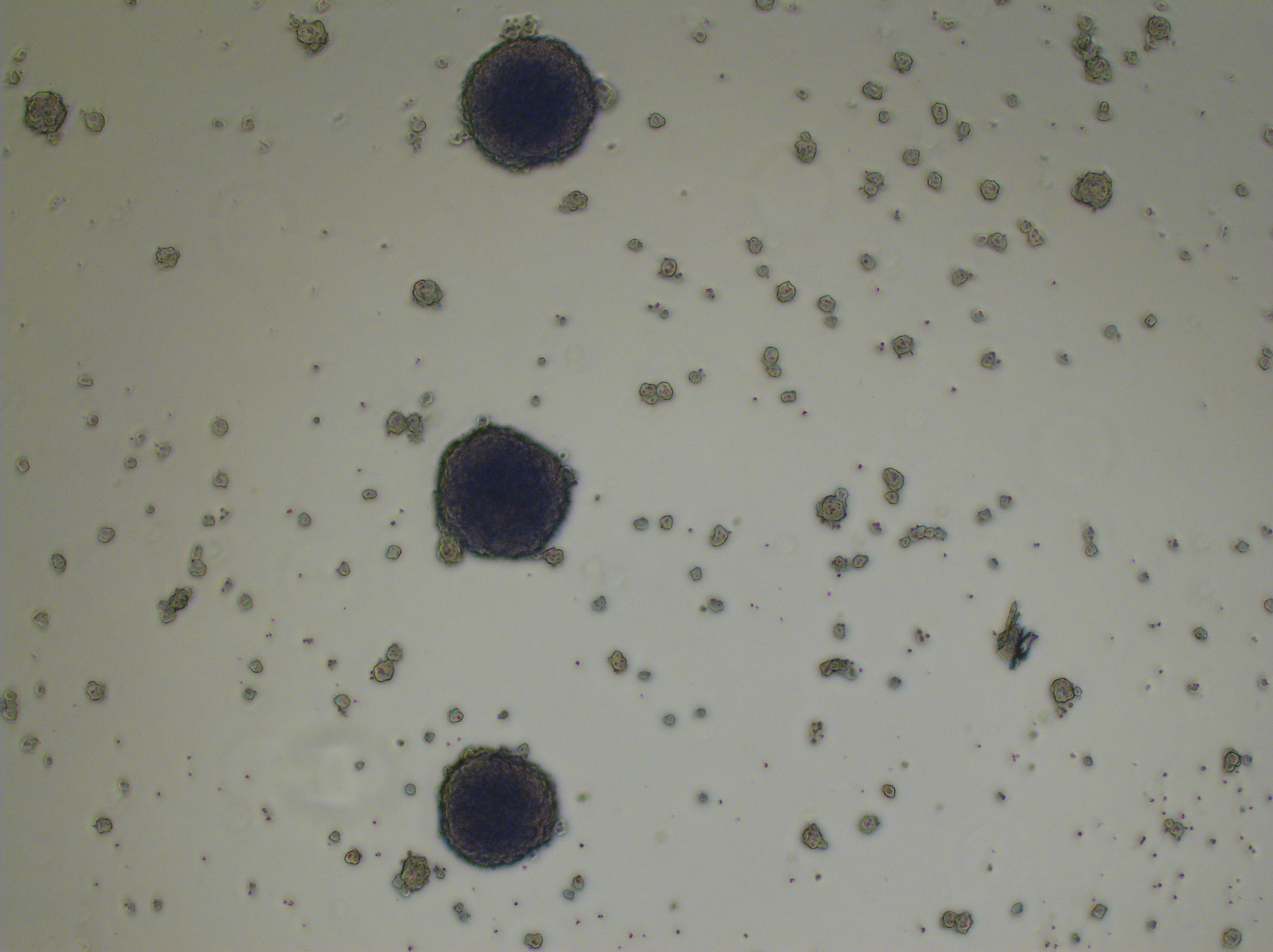

Supplement: S1 File — (ZIP) [file pone.0243812.s001.zip › supporting information/figure 5a/LEFT PANEL/40▒╢-3.jpg]

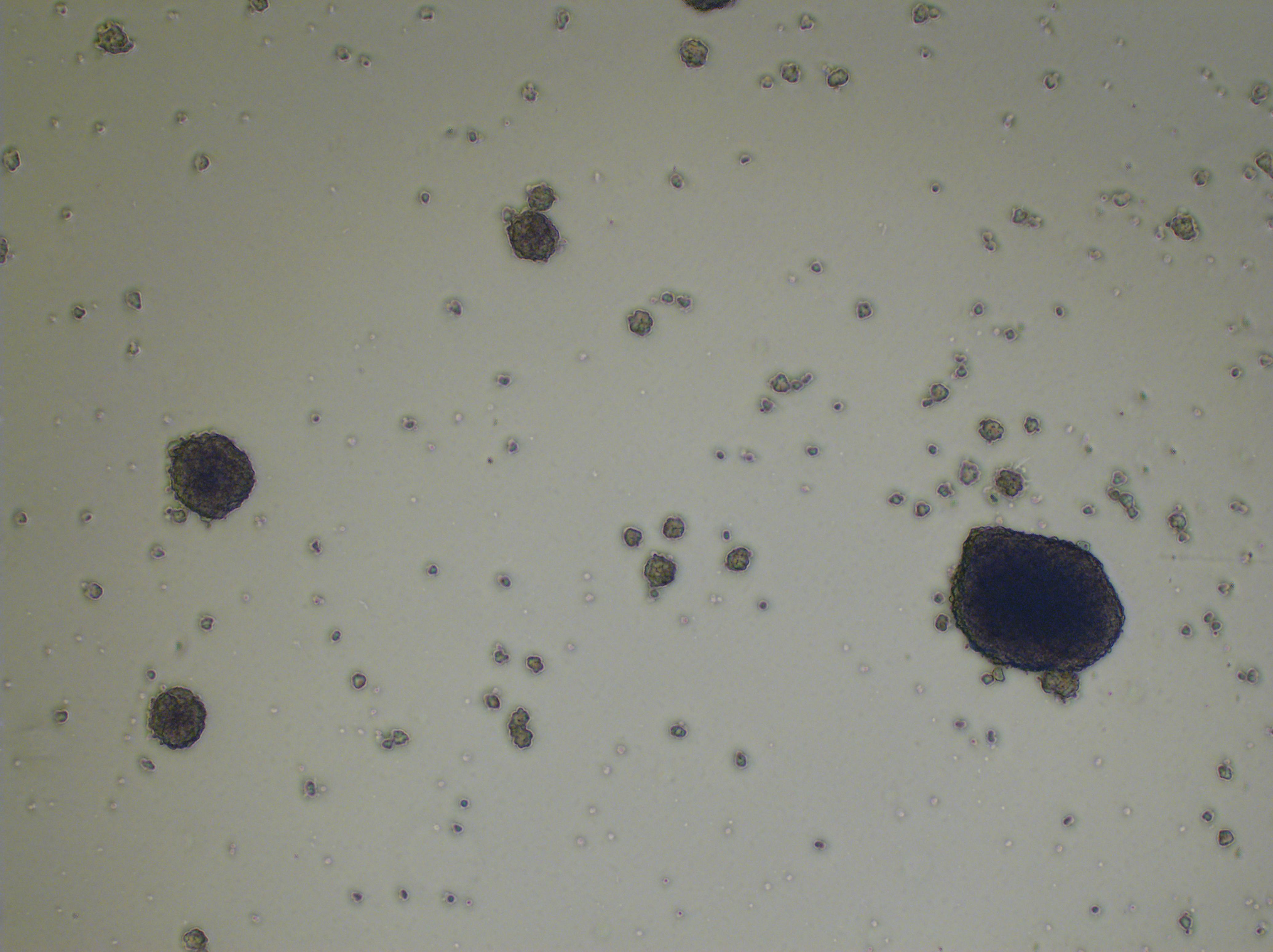

Supplement: S1 File — (ZIP) [file pone.0243812.s001.zip › supporting information/figure 5a/LEFT PANEL/40▒╢-4.jpg]

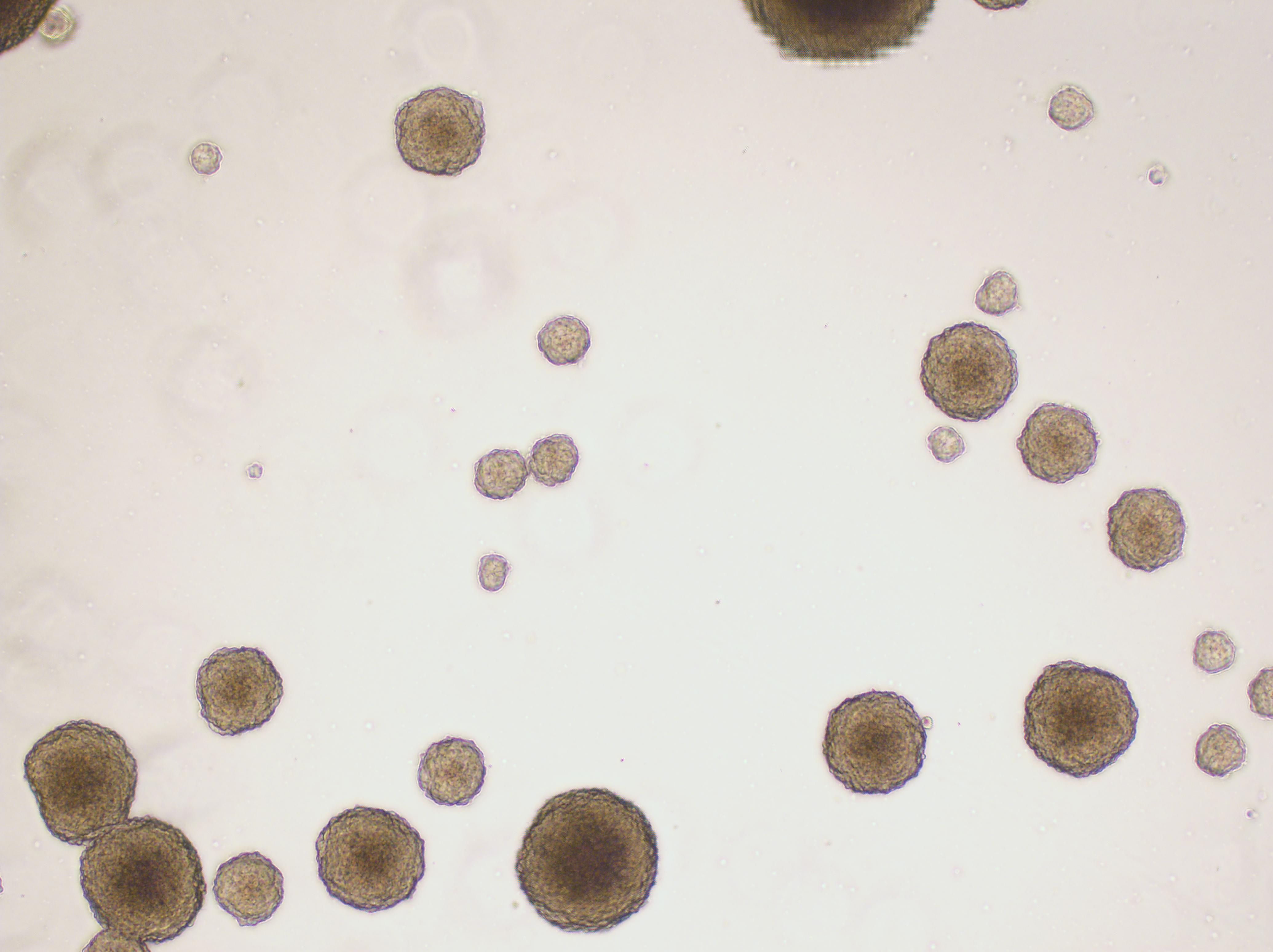

Supplement: S1 File — (ZIP) [file pone.0243812.s001.zip › supporting information/figure 5a/LEFT PANEL/untitled004.jpg]

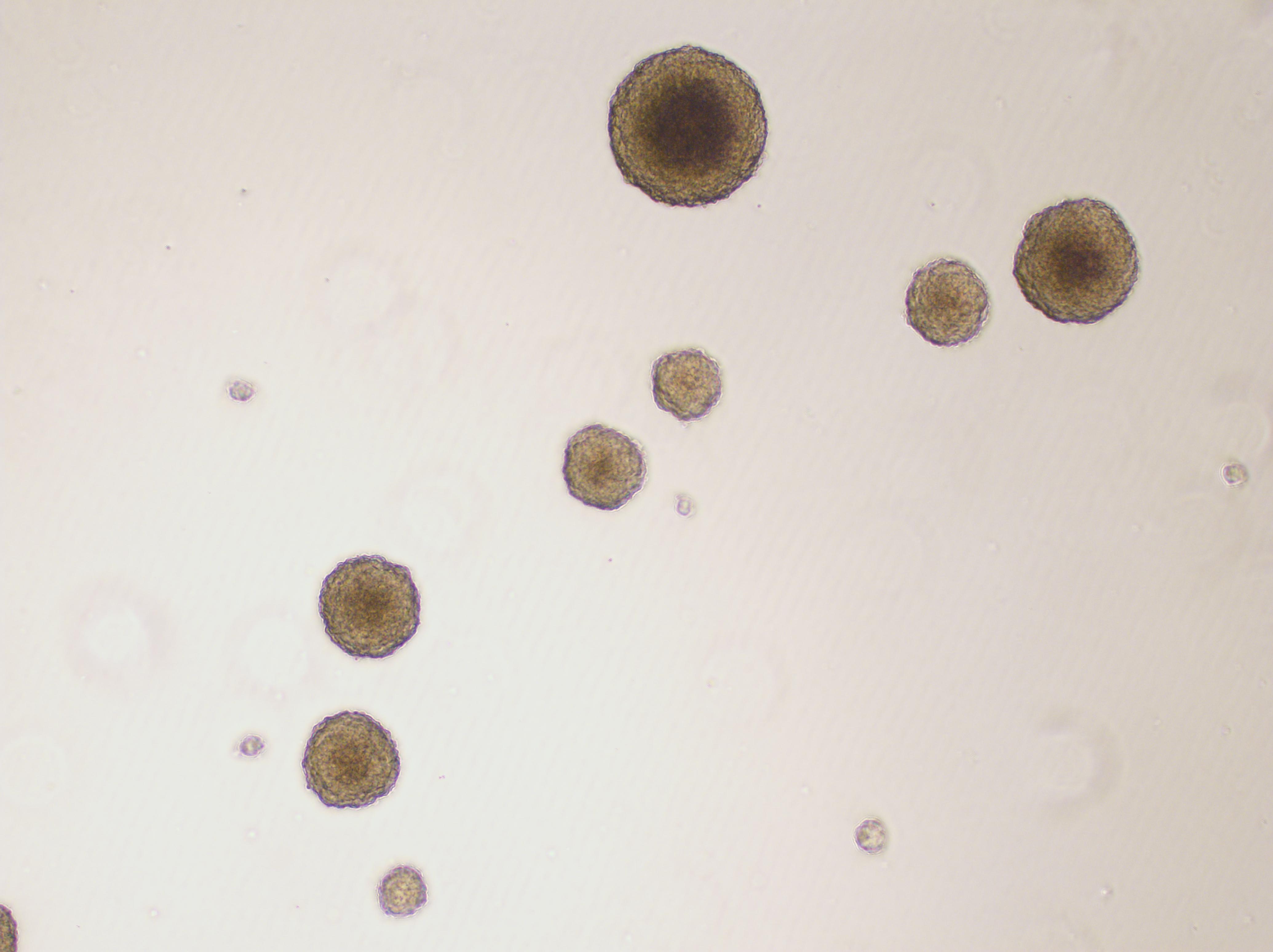

Supplement: S1 File — (ZIP) [file pone.0243812.s001.zip › supporting information/figure 5a/LEFT PANEL/untitled007.jpg]

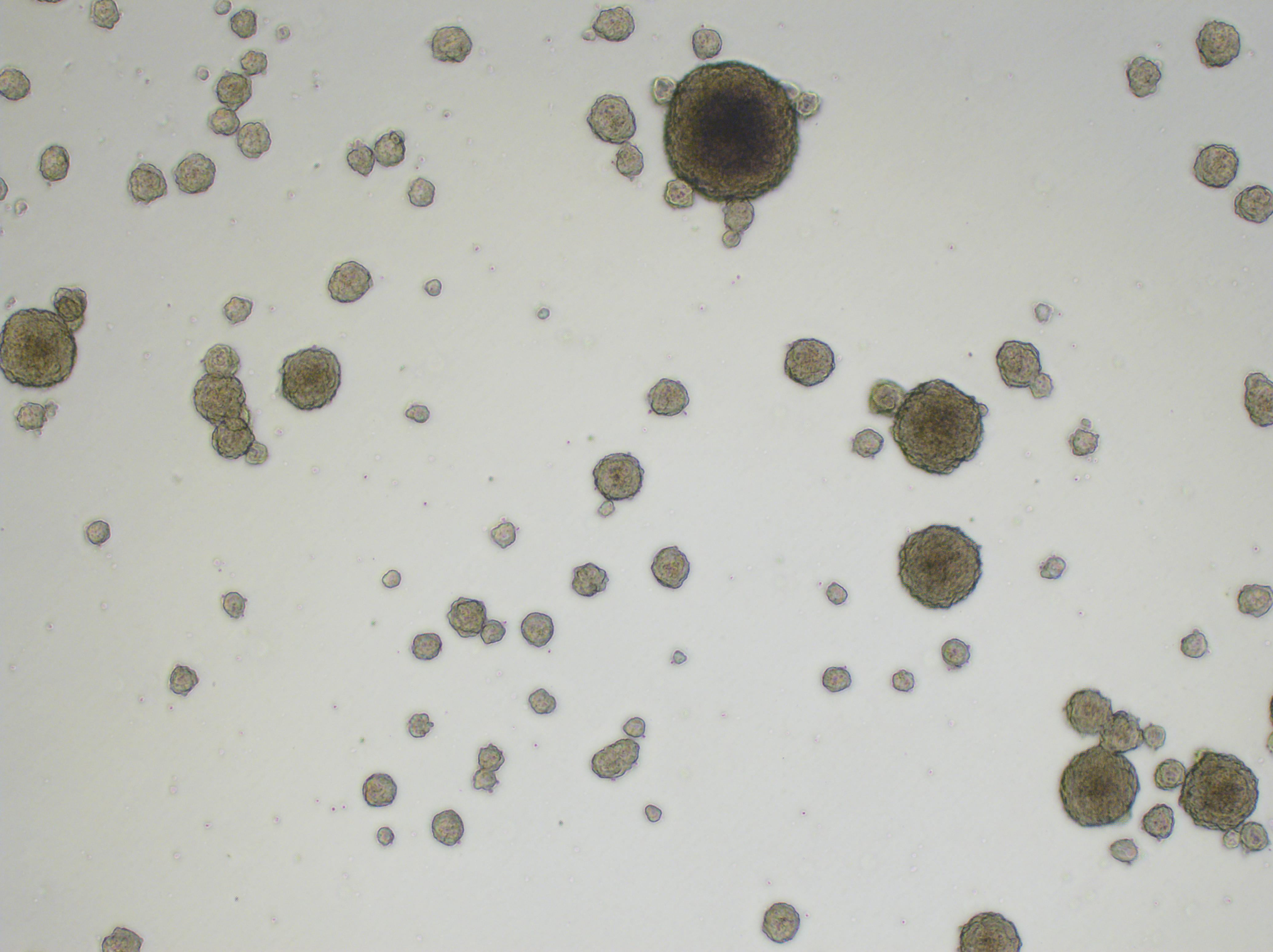

Supplement: S1 File — (ZIP) [file pone.0243812.s001.zip › supporting information/figure 5a/RIGHT PANEL/55.jpg]

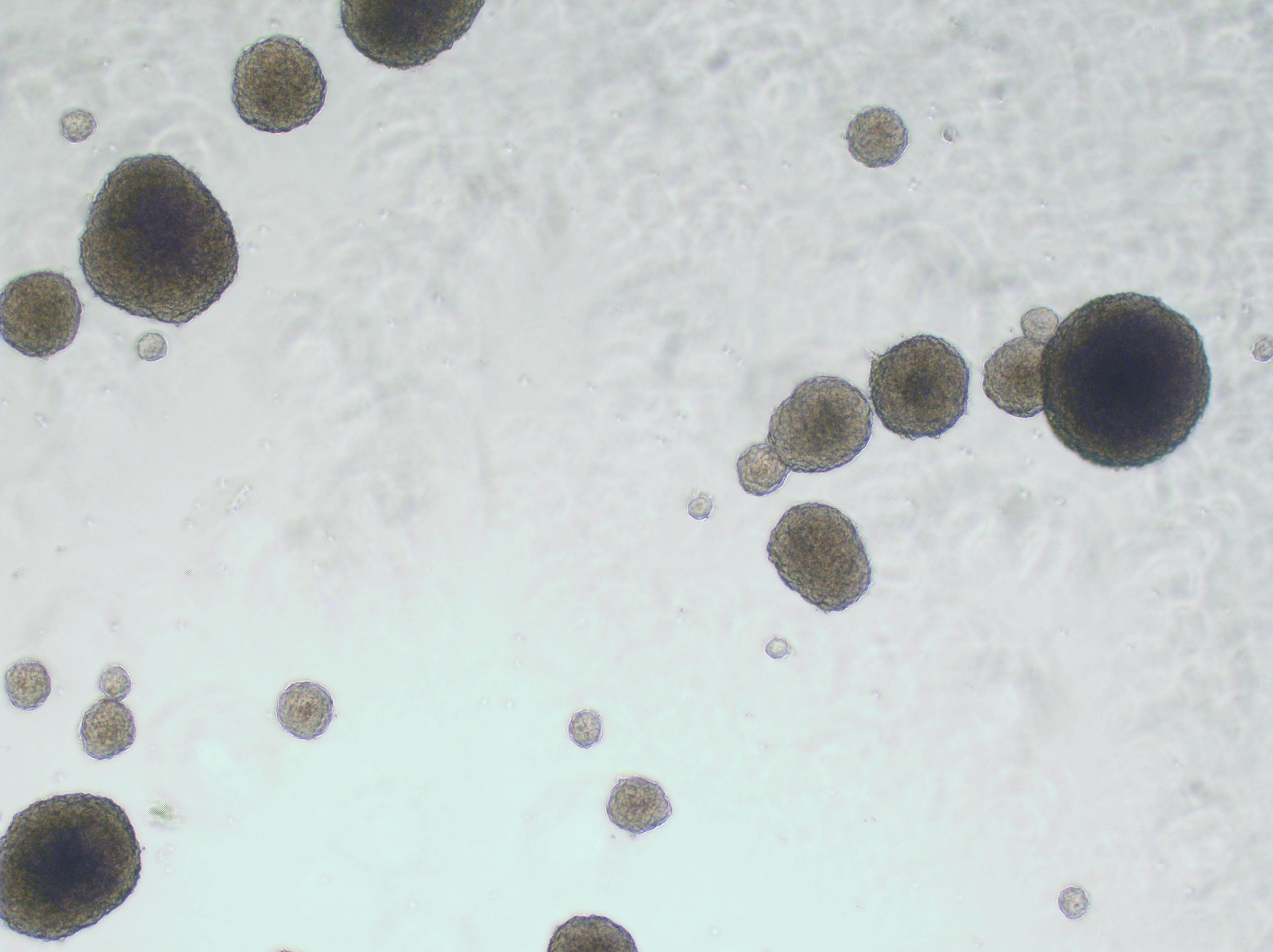

Supplement: S1 File — (ZIP) [file pone.0243812.s001.zip › supporting information/figure 5a/RIGHT PANEL/66.jpg]

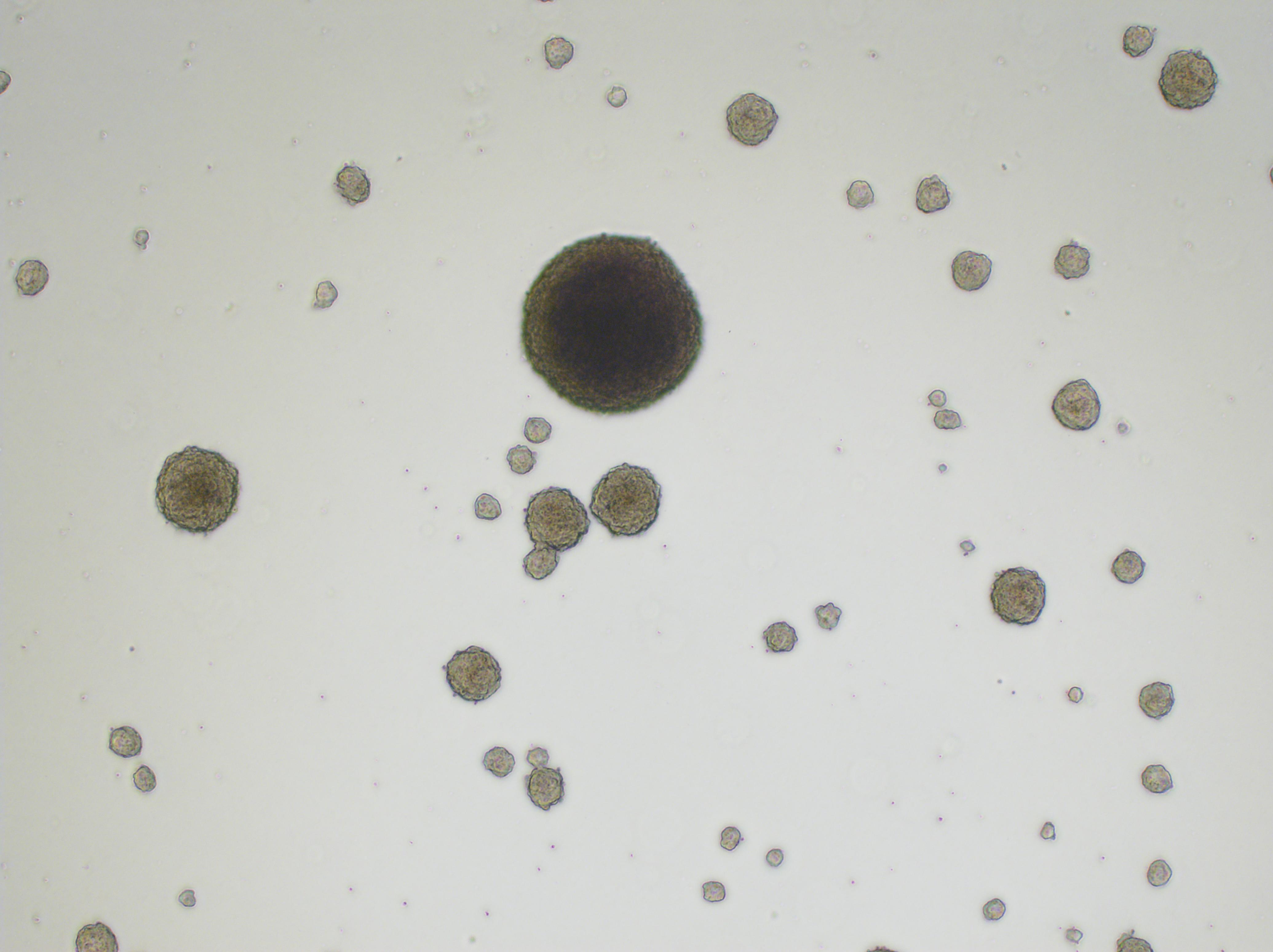

Supplement: S1 File — (ZIP) [file pone.0243812.s001.zip › supporting information/figure 5a/RIGHT PANEL/untitled001.jpg]

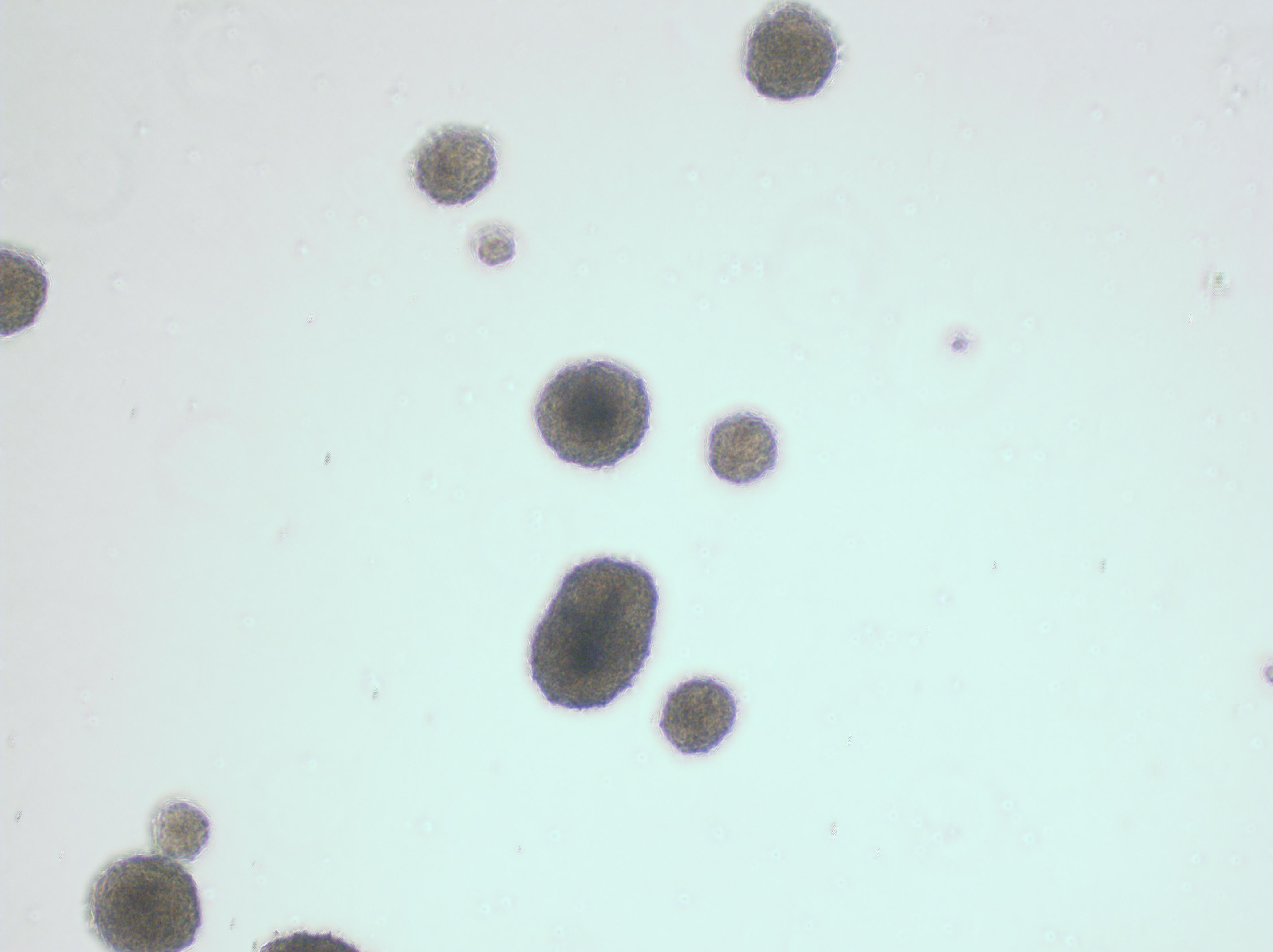

Supplement: S1 File — (ZIP) [file pone.0243812.s001.zip › supporting information/figure 5a/RIGHT PANEL/untitled007.jpg]

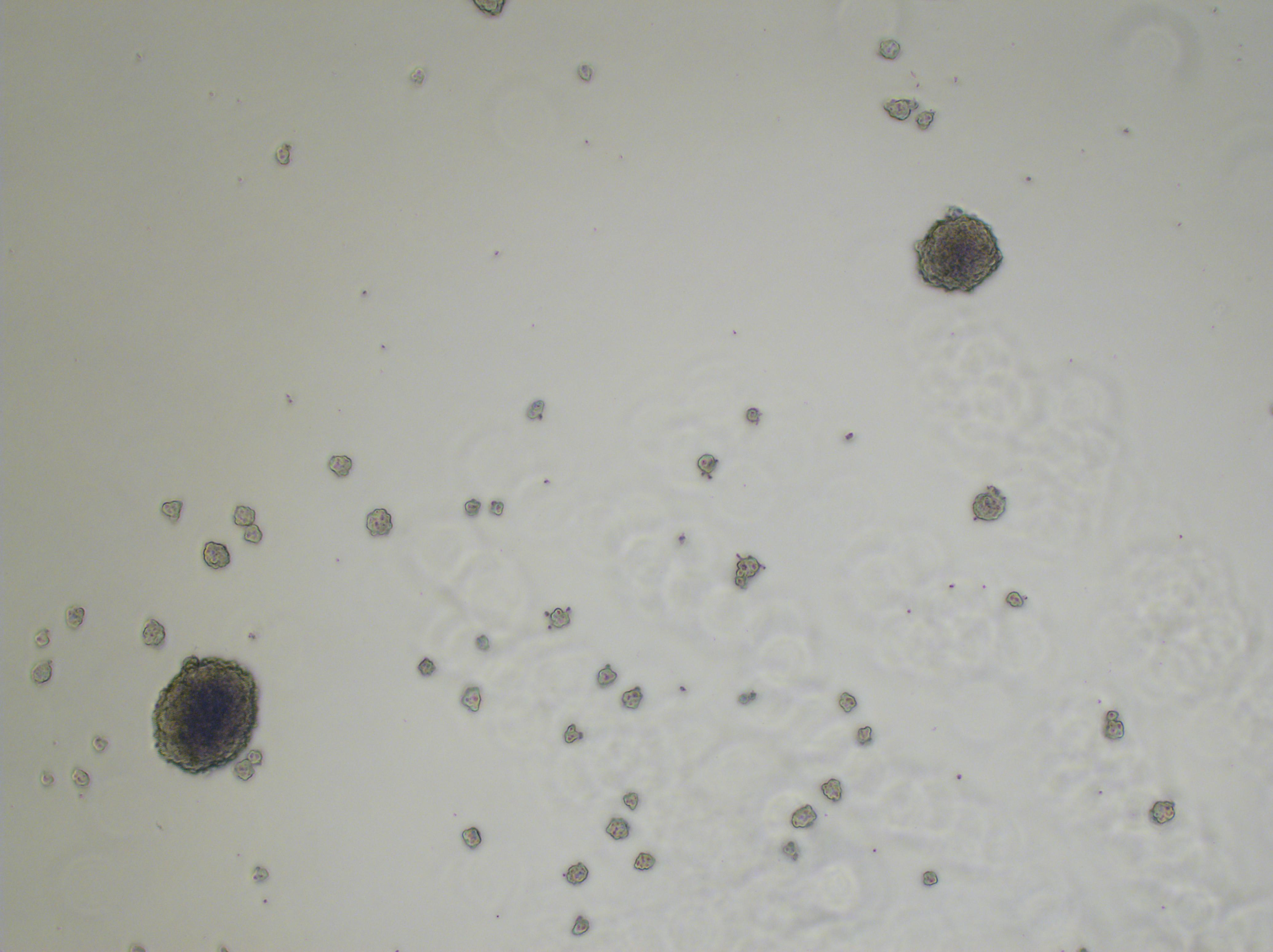

Supplement: S1 File — (ZIP) [file pone.0243812.s001.zip › supporting information/figure 5a/RIGHT PANEL/untitled009.jpg]

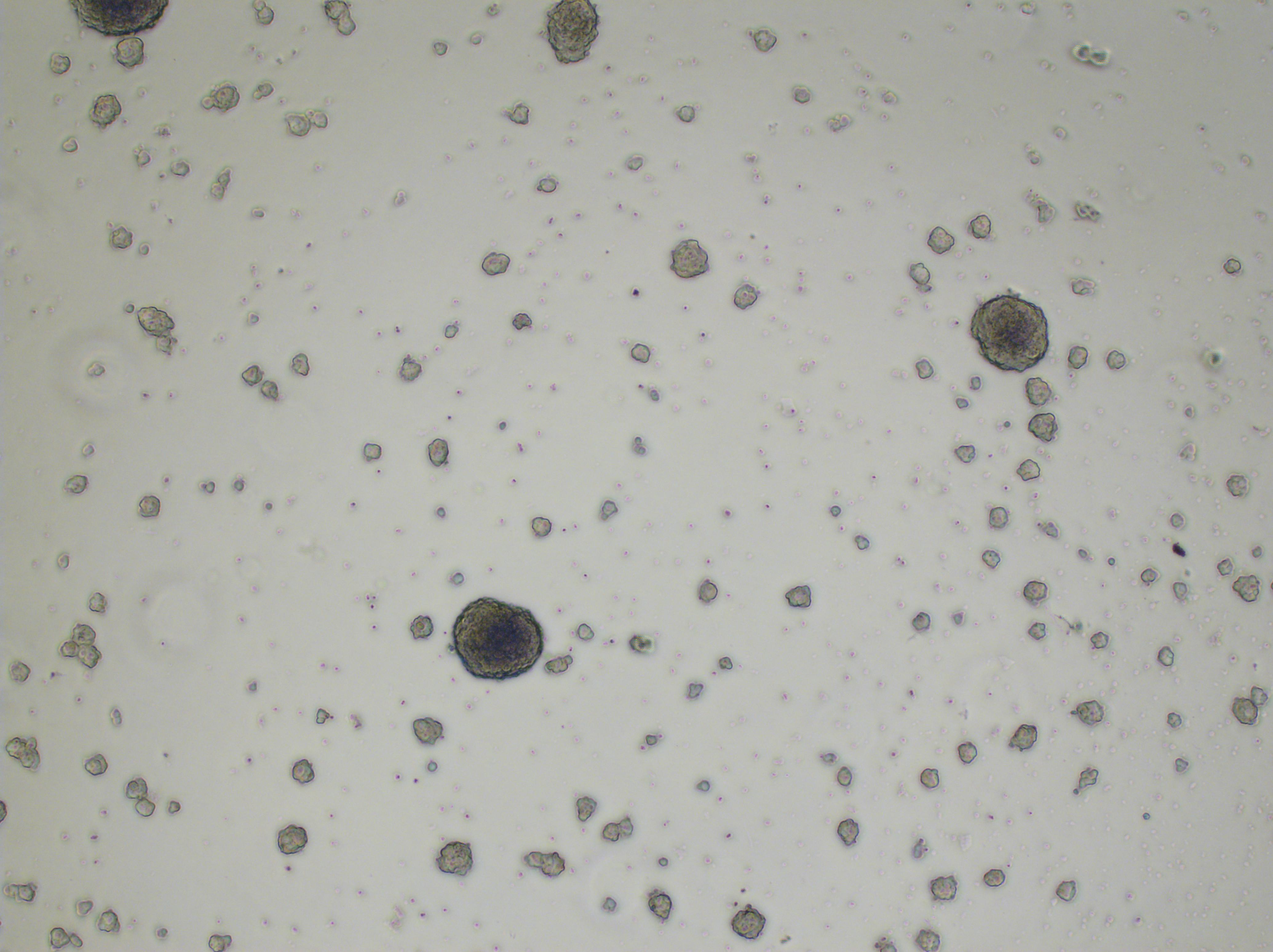

Supplement: S1 File — (ZIP) [file pone.0243812.s001.zip › supporting information/figure 5a/RIGHT PANEL/untitled010.jpg]

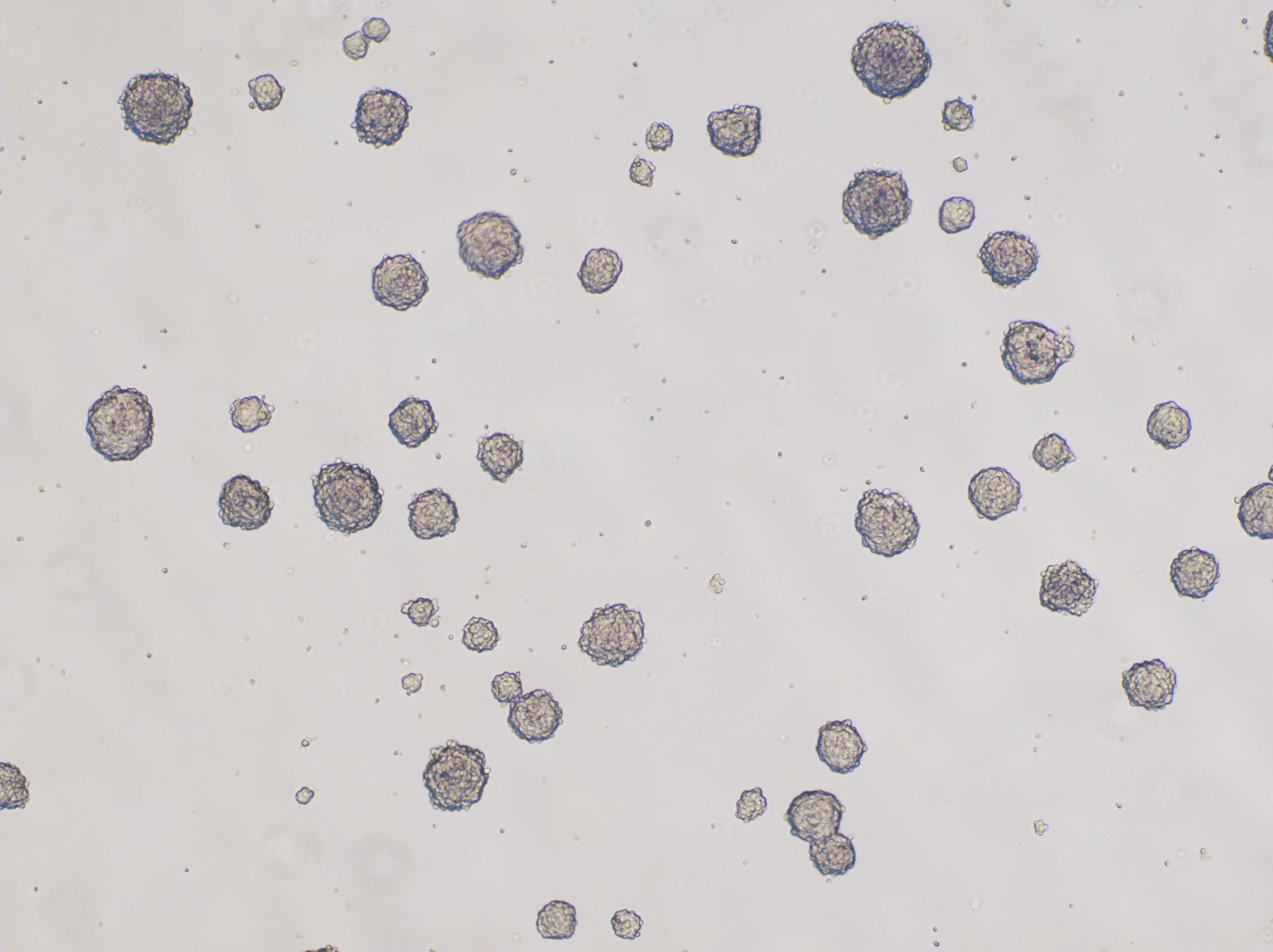

Supplement: S1 File — (ZIP) [file pone.0243812.s001.zip › supporting information/figure 7a/1.jpg]

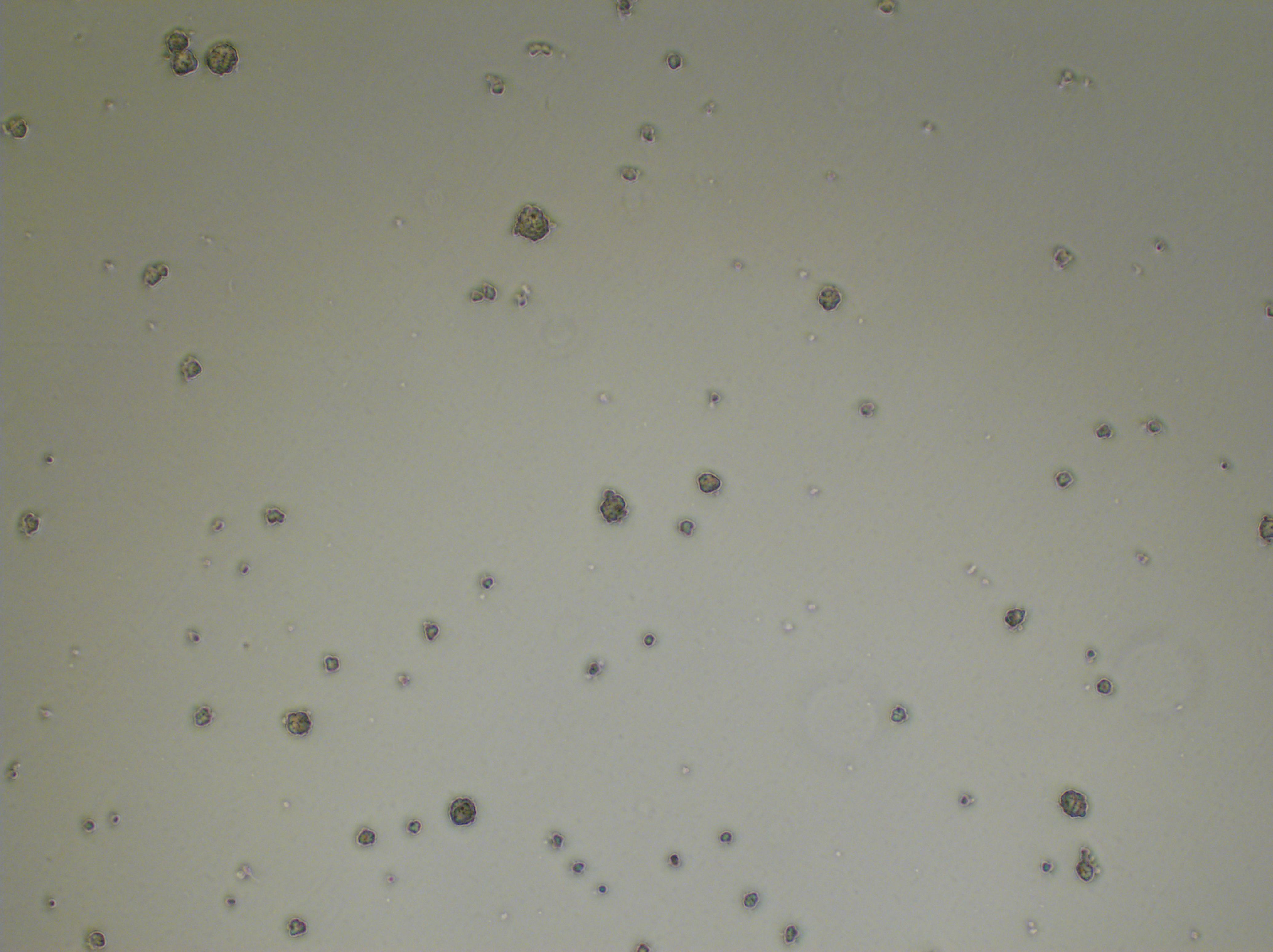

Supplement: S1 File — (ZIP) [file pone.0243812.s001.zip › supporting information/figure 7a/2.jpg]

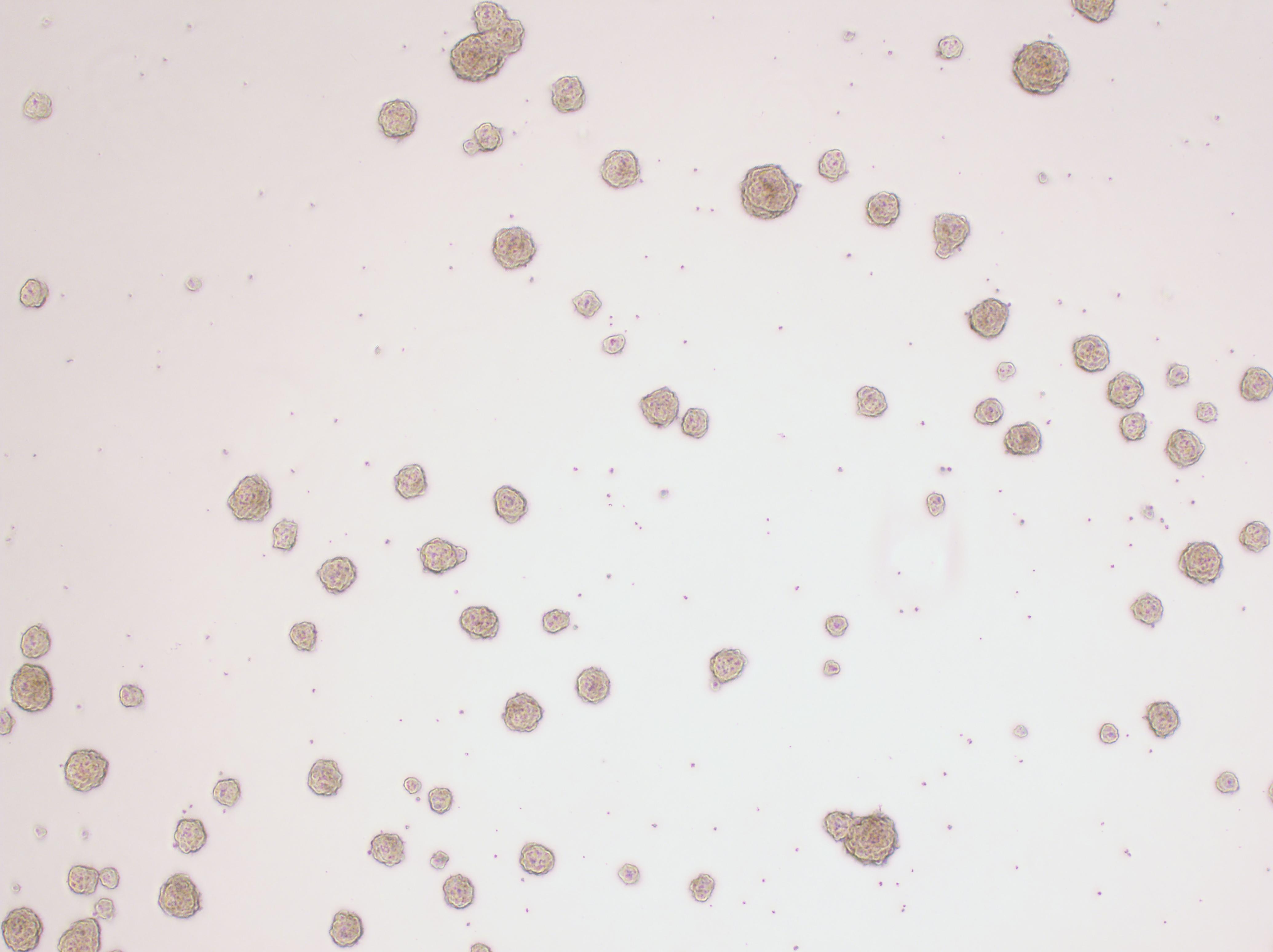

Supplement: S1 File — (ZIP) [file pone.0243812.s001.zip › supporting information/figure 7a/3.jpg]

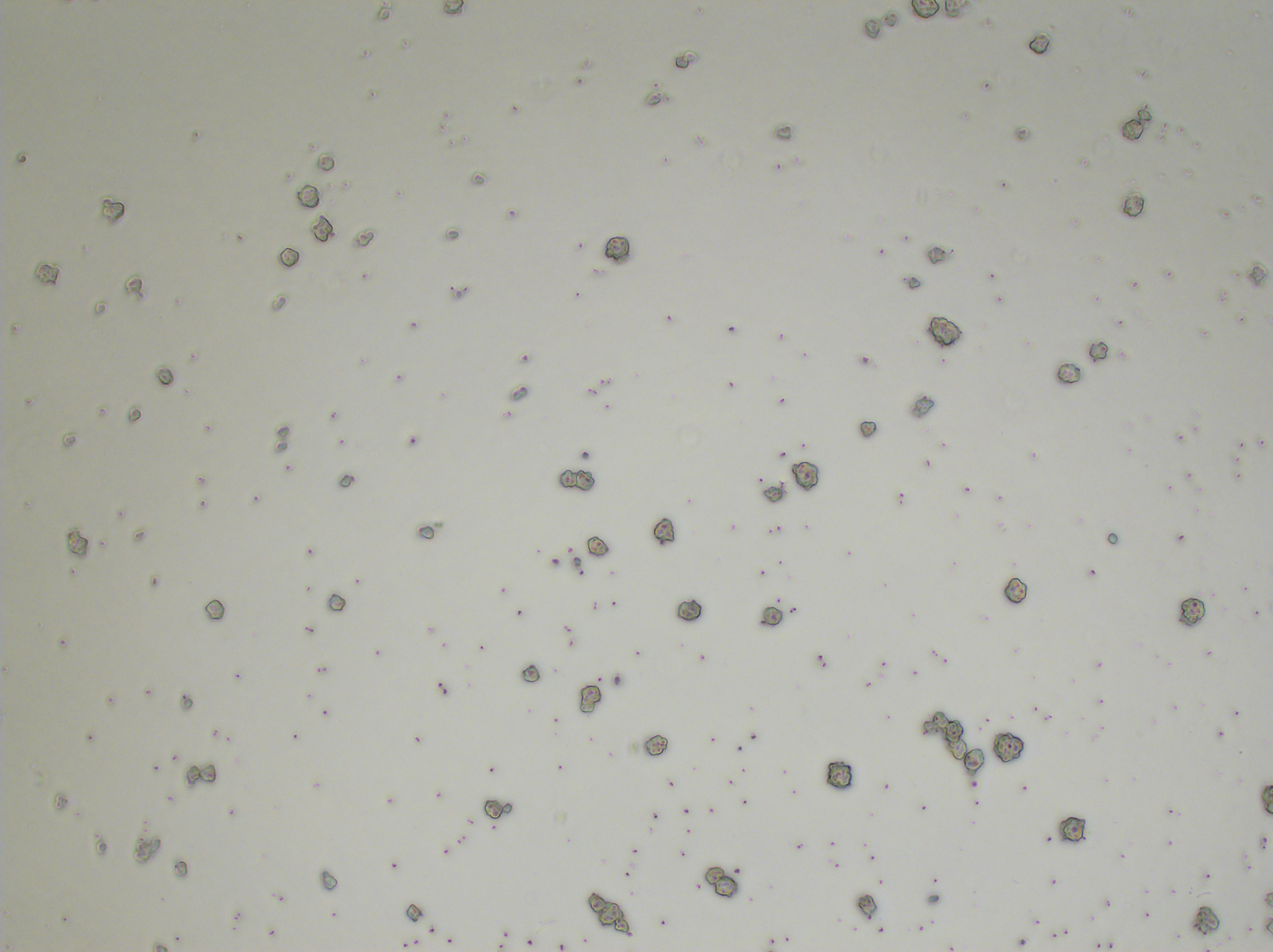

Supplement: S1 File — (ZIP) [file pone.0243812.s001.zip › supporting information/figure 7a/4.jpg]

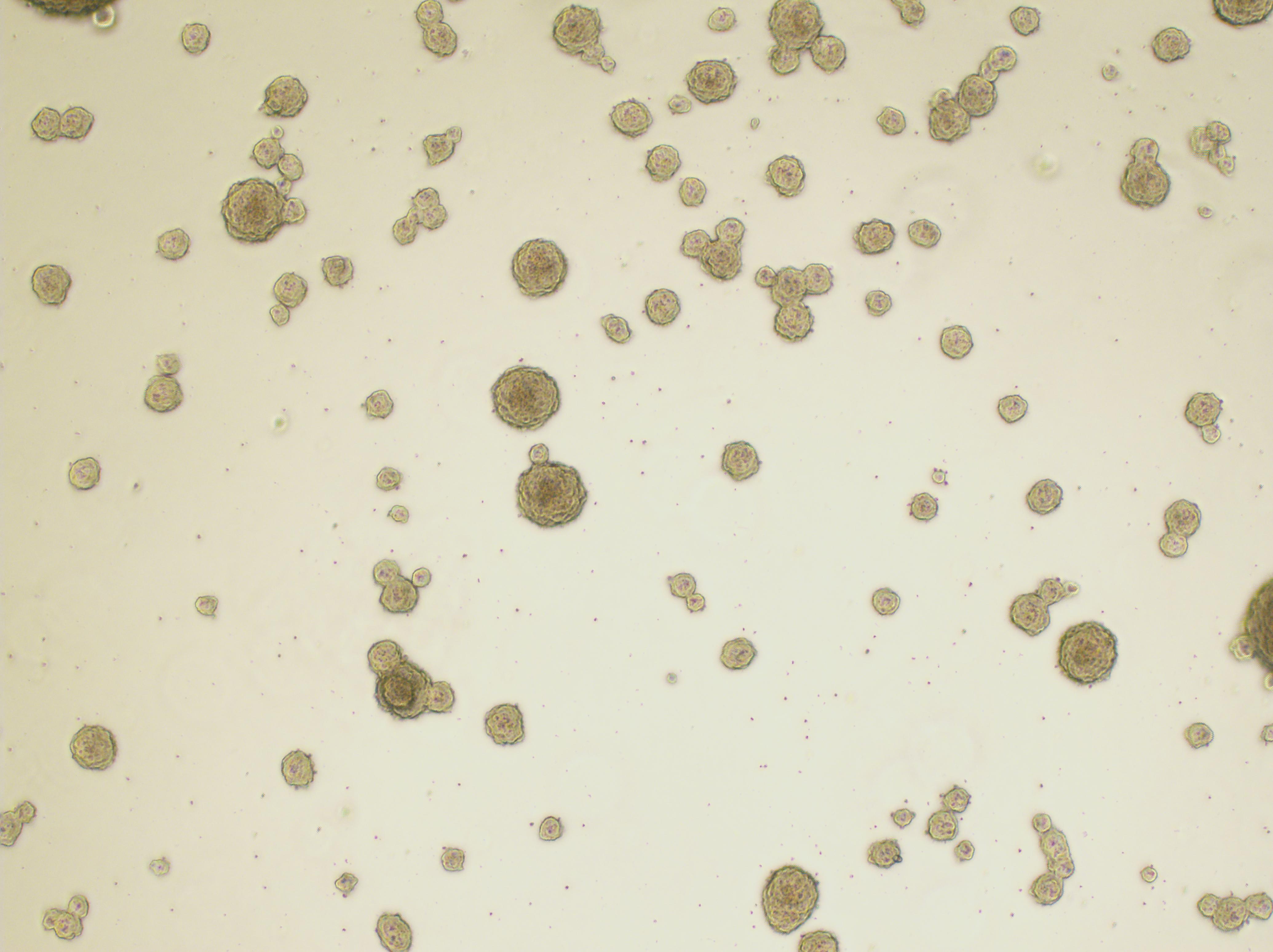

Supplement: S1 File — (ZIP) [file pone.0243812.s001.zip › supporting information/figure 7a/5.jpg]

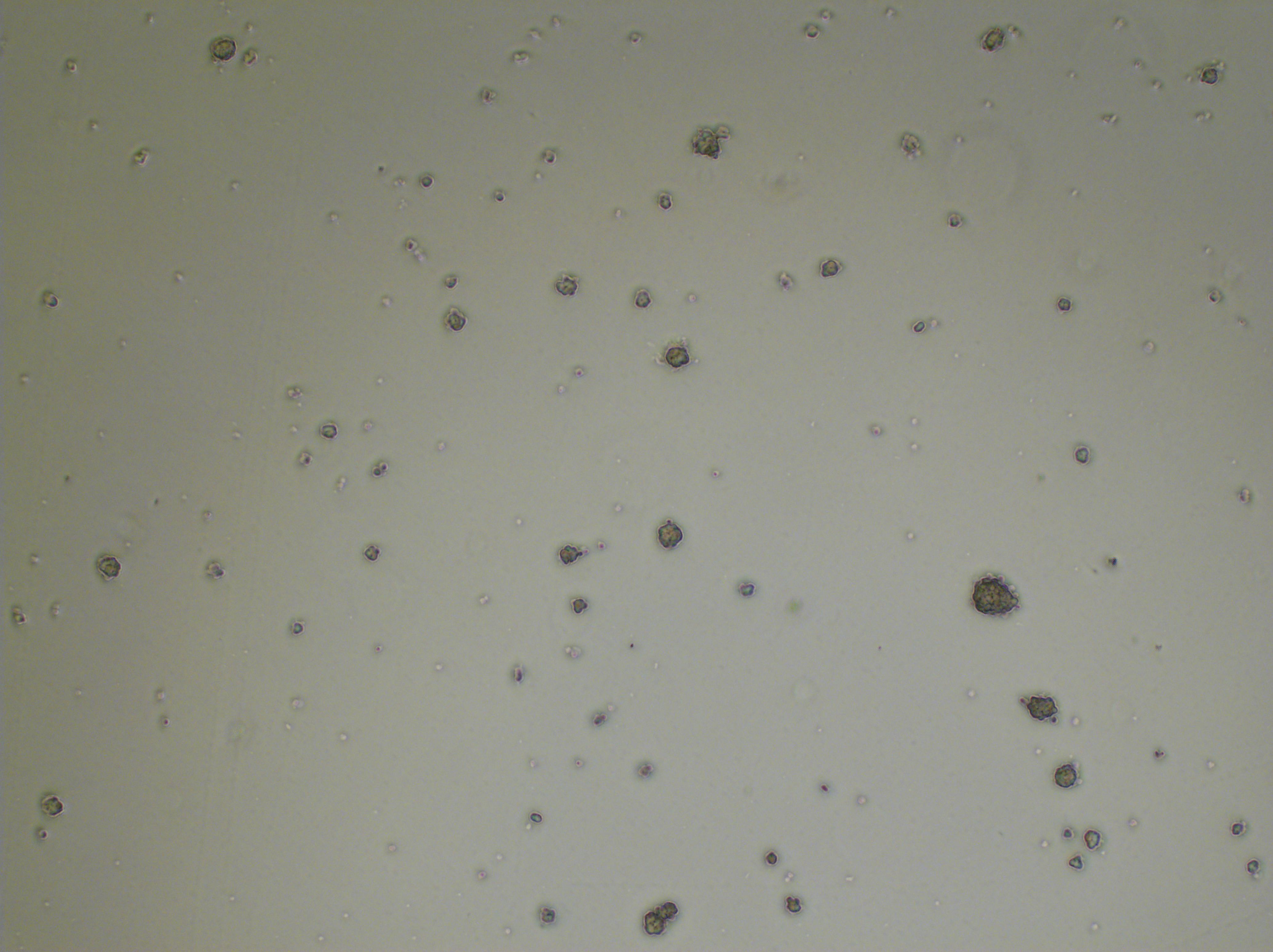

Supplement: S1 File — (ZIP) [file pone.0243812.s001.zip › supporting information/figure 7a/6.jpg]

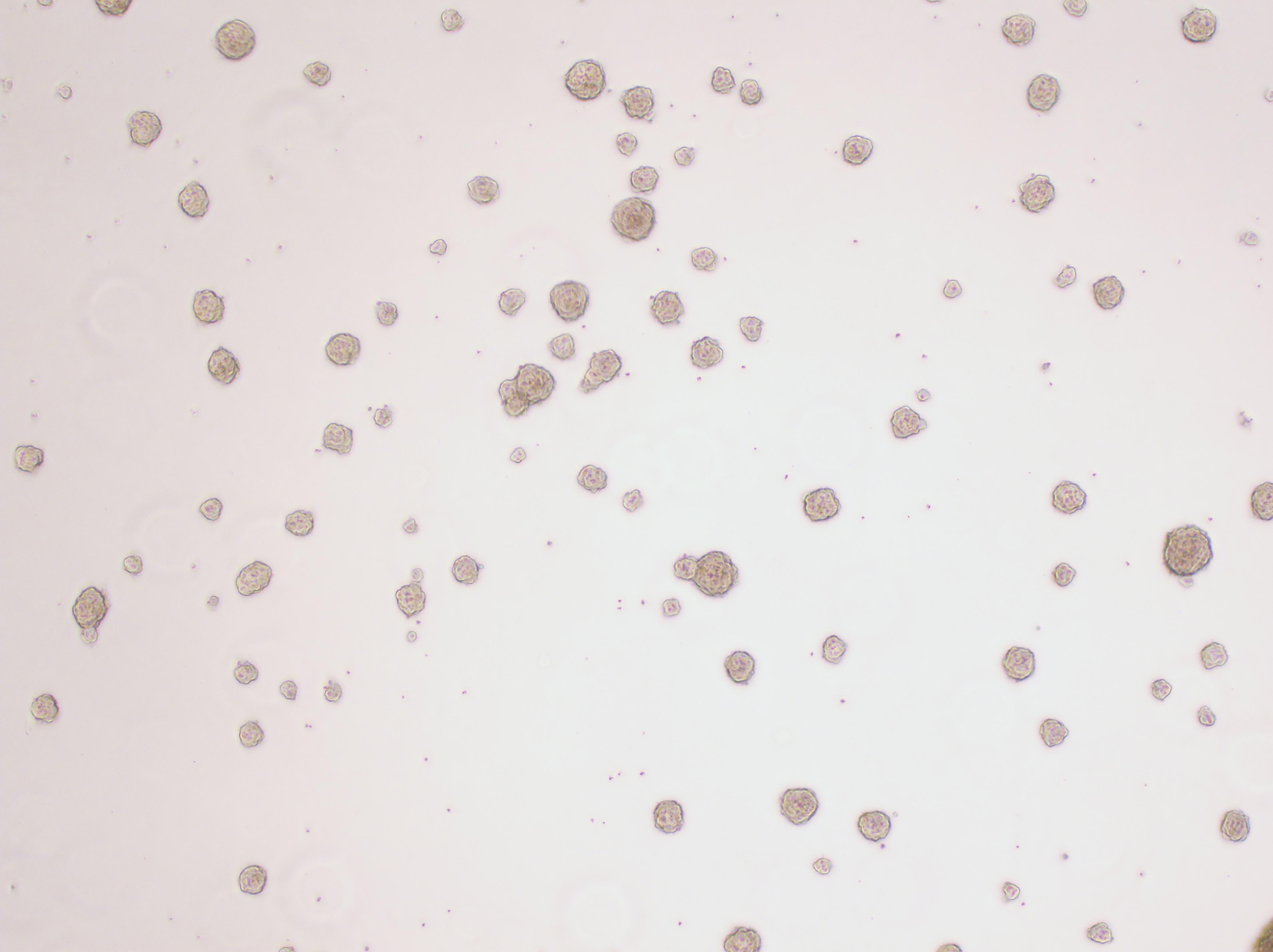

Supplement: S1 File — (ZIP) [file pone.0243812.s001.zip › supporting information/figure 7a/7.jpg]

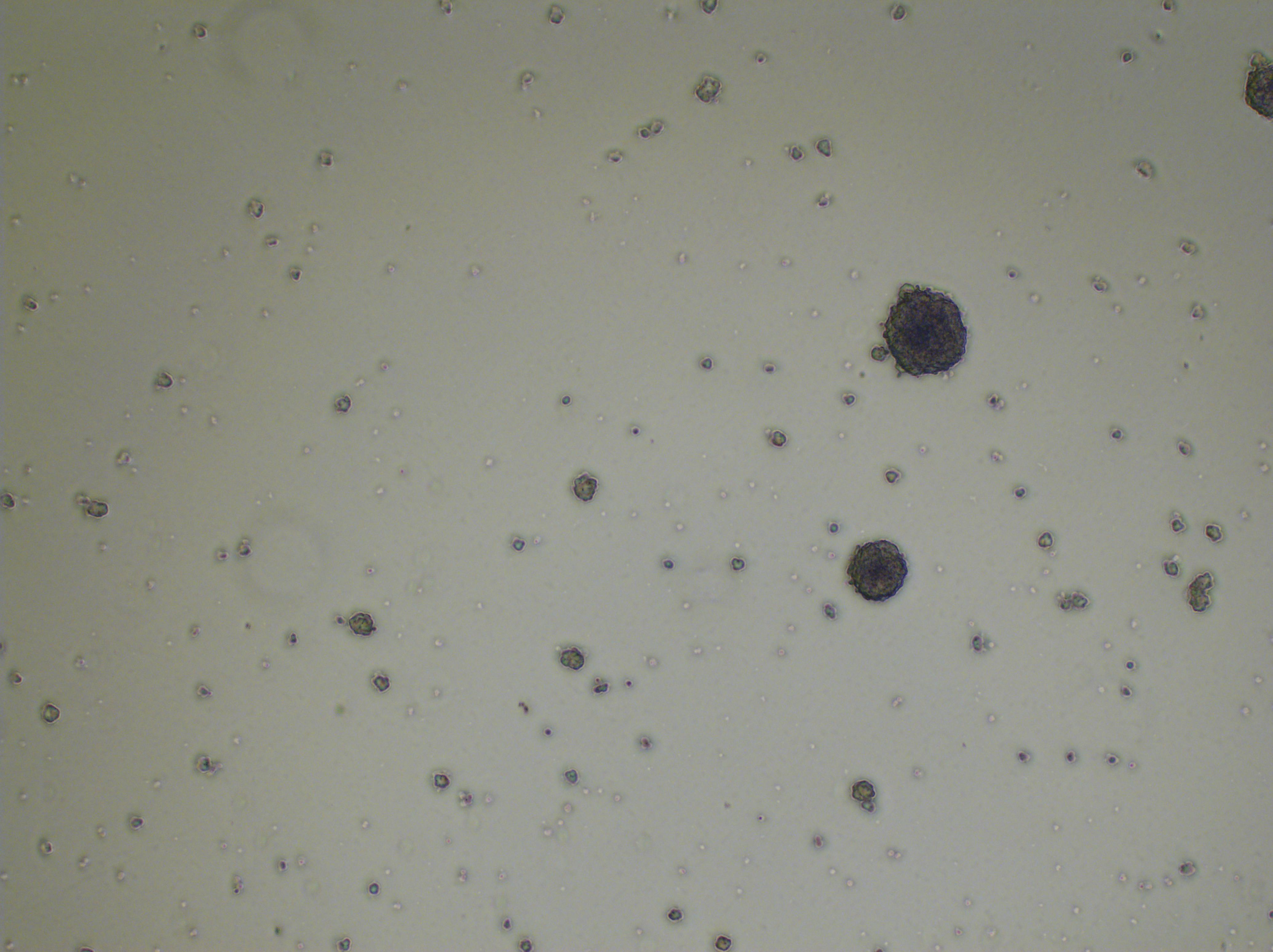

Supplement: S1 File — (ZIP) [file pone.0243812.s001.zip › supporting information/figure 7a/8.jpg]

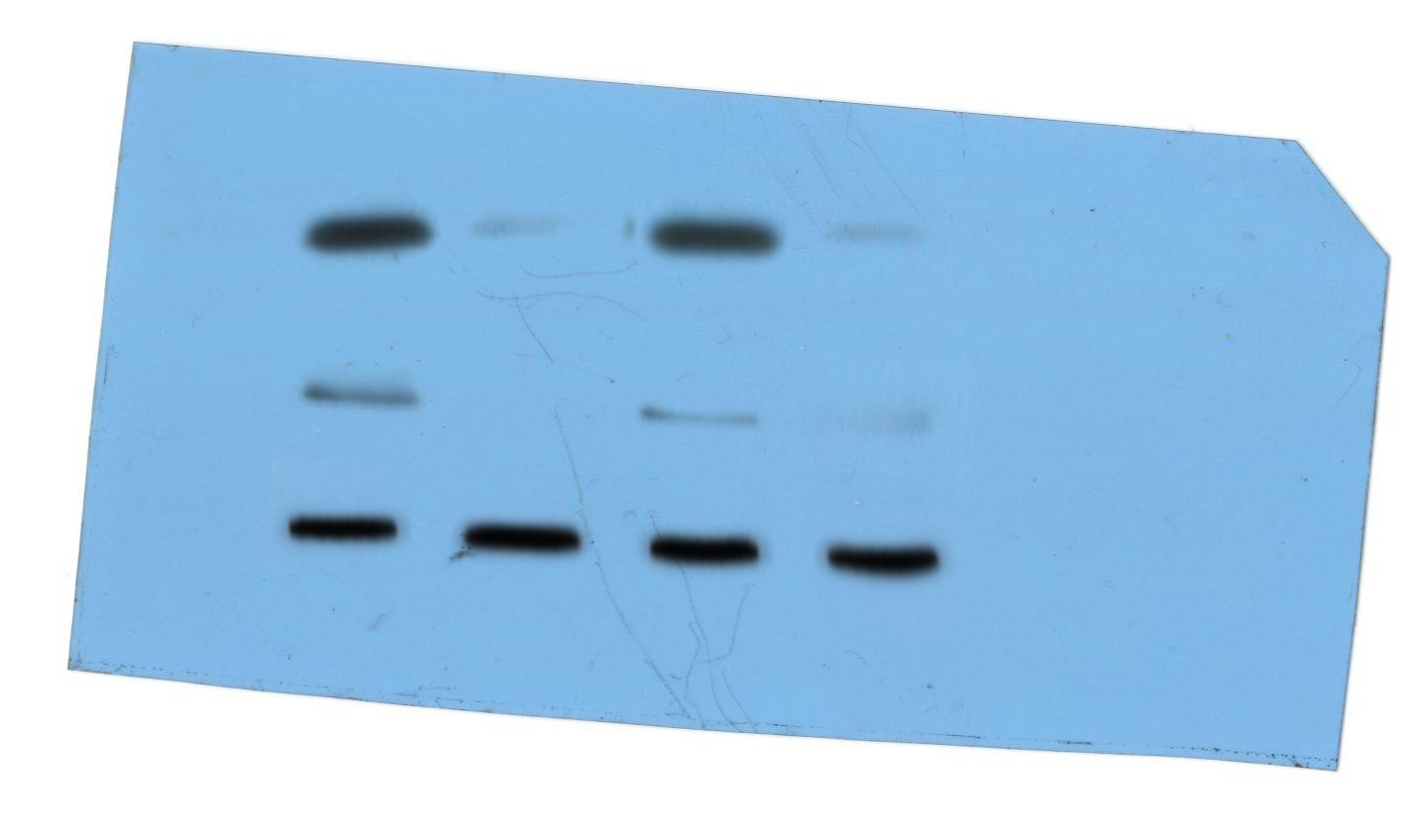

Supplement: S1 File — (ZIP) [file pone.0243812.s001.zip › supporting information/figure 7b/SK-HEP-1 CSCs.jpg]

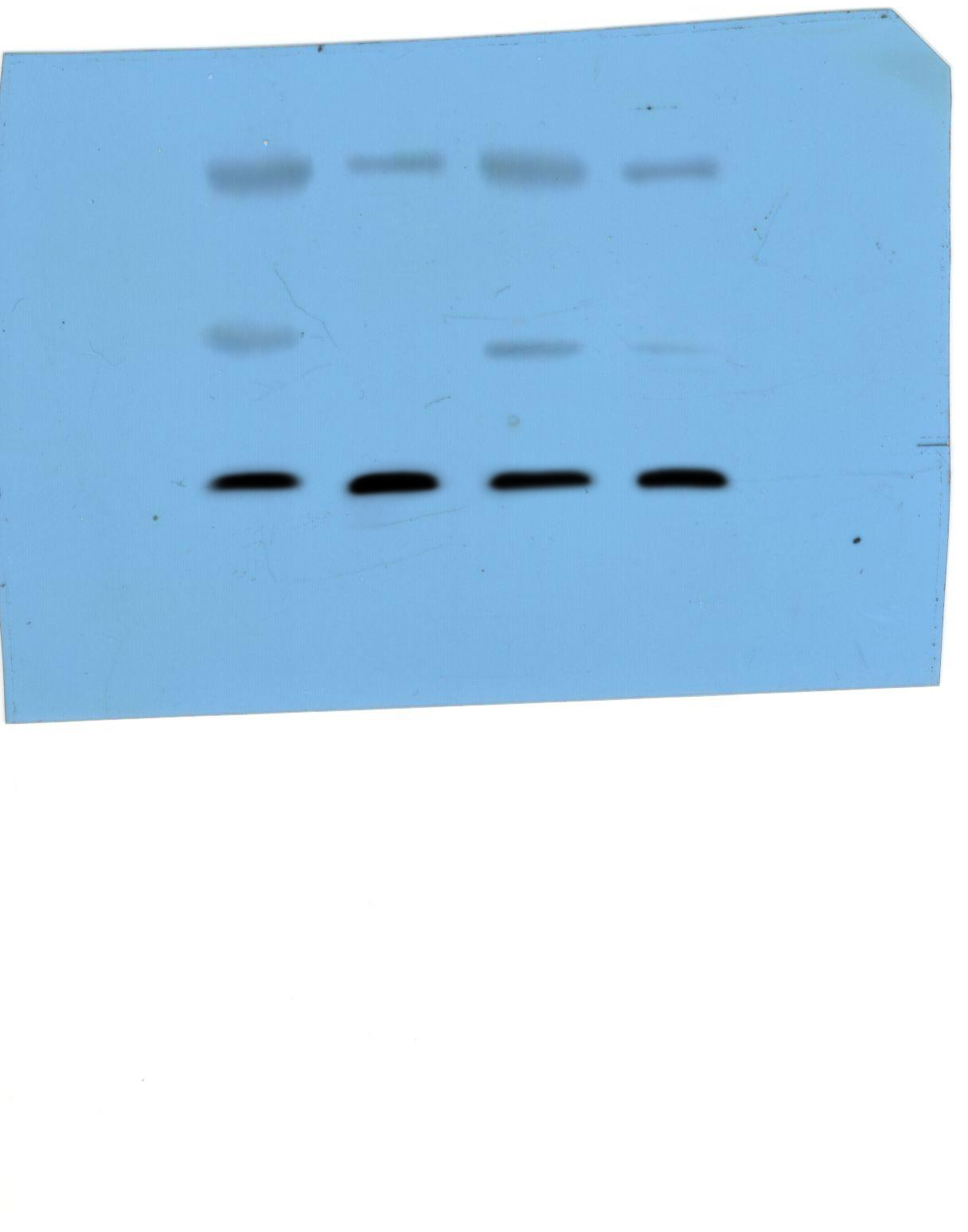

Supplement: S1 File — (ZIP) [file pone.0243812.s001.zip › supporting information/figure 7b/huh-7 CSCs.jpg]
